# An Important Factor Affecting the Supercapacitive Properties of Hydrogenated TiO_2_ Nanotube Arrays: Crystal Structure

**DOI:** 10.1186/s11671-019-3047-2

**Published:** 2019-07-10

**Authors:** Wenyi Li, Wanggang Zhang, Taotao Li, Aili Wei, Yiming Liu, Hongxia Wang

**Affiliations:** 1 Shanxi Key Laboratory of Advanced Magnesium-based Materials, College of Materials Science and Engineering, Taiyuan University of Technology, Taiyuan, 030024 Shanxi China; 2 Shanxi Academy of Analytical Sciences, Taiyuan, 030006 China

**Keywords:** <001> orientation, Rutile/anatase partial transformation, Supercapacitive properties, Hydrogenated TiO_2_ nanotube arrays

## Abstract

Employing a suitable crystal structure can significantly modify the electrochemical performances of materials. Herein, hydrogenated TiO_2_ nanotube arrays with <001> orientation and different rutile/anatase ratio were fabricated via anodisation, high-temperature annealing and electrochemical hydrogenation. The crystal structure was determined by TEM and X-ray diffraction pattern refinement of whole powder pattern fitting. Combined with the model of anatase to rutile transformation and the characterisation of crystal structure, the effect of phase transition on the super capacitive properties of <001> oriented hydrogenated TiO_2_ nanotube arrays was discussed. The results suggested that the anatase grains were characterised by orientation in <001> direction with plate crystallite and stacking vertically to the substrate resulting in excellent properties of electron/ion transport within hydrogenated TiO_2_ nanotube arrays. In addition, the specific capacitance of <001> oriented hydrogenated TiO_2_ could be further improved from 20.86 to 24.99 mF cm^−2^ by the partial rutile/anatase transformation due to the comprehensive effects of lattice disorders and rutile, while the good rate performance and cyclic stability also retained.

**Electronic supplementary material:**

The online version of this article (10.1186/s11671-019-3047-2) contains supplementary material, which is available to authorized users.

## Introduction

TiO_2_ is an important type of multifunctional semiconductor materials. Owing to the advantages including low cost, nontoxicity, facile processability and excellent stability [1–5], it has been drawn much attention in light harvesting device applications, such as solar cells [6, 7], photodetectors [8–11], photoelectrochemical water splitting [12, 13] and photocatalysis [14]. In recent decades, inheriting all the typical features of TiO_2_ materials and displaying not only the relatively high specific area but also straight pathway for carrier transmission along the axial direction, TiO_2_ nanomaterial, especially TiO_2_ nanotube arrays (TNAs) fabricated by anodic oxidation, was considered as a promising candidate for supercapacitor electrode with high power density, long-term cycling stability and fast charging/discharging ability [5, 15–20]. However, due to the wide band gap and consequent low concentration of carrier, the extensive application of TNAs in supercapacitor field was limited by the poor conductivity of pristine TiO_2_(10^−5^~10^−2^ S m^−1^) [21]. Various approaches have been carried out to enhance the conductivity of TNAs, which involved introducing other materials with a special morphology and doping with non-metal ions [22]. Among those approaches, hydrogenation gave researchers a new horizon. The carrier concentration within TiO_2_ can be significantly increased by hydrogenation, thus enhancing the conductivity of TiO_2_ [23–25]. The proper microstructure, including bonding structure, heterostructure, junction, phase composition and orientation, is necessary for efficient diffusion of the carrier with high density, which will ensure good electrochemical performance [26–34]. The phase composition and orientation are the two most crucial microstructure parameters affecting the carrier transmission, which can be modified to improve the electrochemical properties of TiO_2_ [35–37]. In contrast with photocatalytic applications, in which it has been reported that the rutile/anatase composite materials and the anatase TNAs with dominant {001} facets were both more efficient than anatase counterparts [38–41], however, in hydrogenated TNAs case, detailed investigation of such promising configurations is limited. Most of the works focused on anatase hydrogenated TiO_2_ nanotube arrays (H@TNAs) while ignored the effects of the TiO_2_ crystal structure on the electrochemical performance of H@TNAs [5, 19, 42–45]. Inspired by these works mentioned above and considering the potential applications of TiO_2_-based materials in supercapacitors, it is of great significance to clarify the interrelationship between crystal structure (orientation and phase composition) and the electrochemical performance of H@TNAs.

Herein, highly ordered TNAs with <001> orientation were prepared by a two-step anodisation and a subsequent annealing process. The phase content of TNAs can be adjusted by the annealing temperature and the holding time. Then, the as-prepared TNAs were hydrogenated by a facile electrochemical hydrogenation process. Subsequently, various microstructural and electrochemical characterisations were conducted to investigate the interrelationship between the crystal structure and the electrochemical performances.

## Methodology

### Materials

The detailed information of raw materials involved in the experiment is listed in Table 1.

**Table 1:** Raw materials involved in the experiment

Materials	Purity	Provider
Commercial pure titanium plates	99.99%	China Research Institute of Nonferrous Metals
NH_4_F	Analytical reagent (≥ 99.7%)	Tianjin Kemiou Chemical Reagent Co., Ltd.
Na_2_SO_4_	Analytical reagent (≥ 99.7%)	Tianjin Kemiou Chemical Reagent Co., Ltd.
Ethylene glycol	Analytical reagent (≥ 99.7%)	Tianjin Kemiou Chemical Reagent Co., Ltd.
Acetone	Chemical reagent (≥ 99.5%)	Tianjin Kemiou Chemical Reagent Co., Ltd.
Ethanol	Chemical reagent (≥ 99.5%)	Tianjin Kemiou Chemical Reagent Co., Ltd.
Deionised water	–	Homemade

### Synthesis of Hydrogenated <001> Oriented TiO_2_ Nanotubes

A two-step anodisaton process was used to prepare TNAs. Commercial pure titanium plates were cut into sheets of 30 × 10 × 0.1 mm^3^. Before anodisation, the titanium sheet was cleaned by sonication sequentially for 30 min in deionised water, 30 min in acetone and finally 30 min in alcohol. The anodising process was carried out at 30 °C, in a two-electrode configuration with a water-glycol solution containing NH_4_F 0.3 g, H_2_O 2 mL and ethylene glycol 98 mL, where the titanium sheet was the working electrode and a platinum sheet is the counter electrode. The titanium sheets were anodised at the condition of electric voltage 50 V, interelectrode distance 2 cm, and anodised time 1 h. Then, the titanium sheet was washed by sonication in deionised water, after which the titanium sheet was anodised again at the same condition to obtain the highly ordered TNAs. TNAs fabricated by anodising process were amorphous [46]. The as-prepared TNAs were heat-treated in a tube furnace to obtain TNAs with different polymorphs. The anatase <001> oriented TNAs (noted as TNAs-1) was annealed at 450 °C for 3 h in an argon atmosphere. The <001> oriented TNAs with different rutile/anatase ratio were annealed at 650 °C for 1 to 3 h and noted as TNAs-2, TNAs-3, and TNAs-4, respectively.

**Fig. 1:**
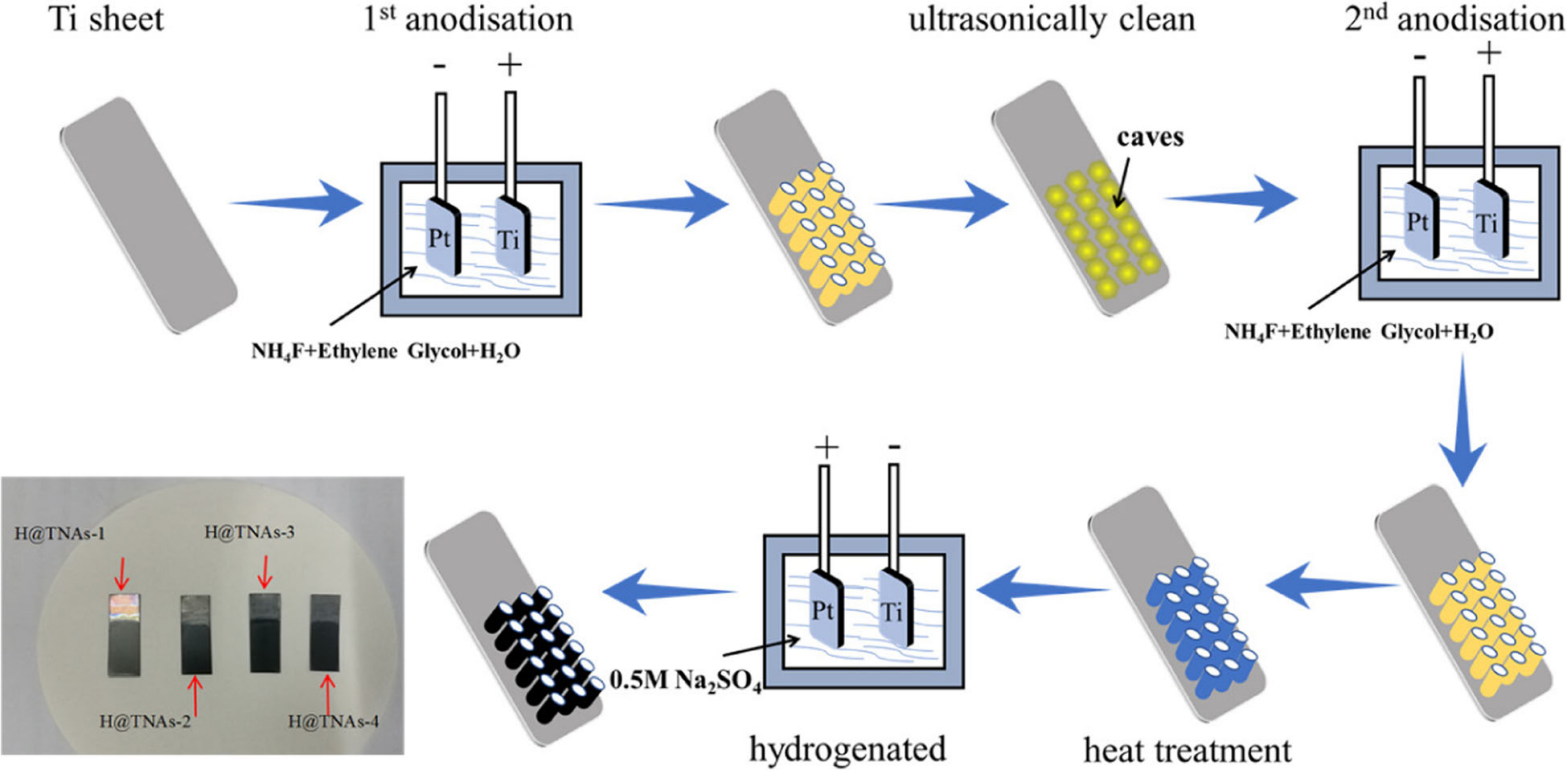
Schematic diagram of preparation and the optical image of as-prepared H@TNAs

The hydrogenation was induced by a simple electrochemical process. The as-heat-treated TNAs were hydrogenated in a two-electrode configuration with a 0.5-M Na_2_SO_4_ solution. The TNAs were employed as a cathode, and a platinum sheet worked as an anode, separately. The distance between the two electrodes was 2 cm, the electric voltage applied was 5 V and the processing time was 30 s. Detailed preparation parameters of the samples were listed in Table 2. The experiment route is illustrated in Fig. 1.

**Table 2:** Preparation parameters of samples

Samples			H@TNAs-1	H@TNAs-2	H@TNAs-3	H@TNAs-4
Anodisation	Electrolyte composition	NH_4_F (g)	0.3	0.3	0.3	0.3
		H_2_O (mL)	2	2	2	2
		Ethylene Glycol (mL)	98	98	98	98
	Applied voltage (V)	First anodisation	50	50	50	50
		Second anodisation	50	50	50	50
	Processing time (h)	First anodisation	1	1	1	1
		Second anodisation	1	1	1	1
Annealing	Heating rate (°C min^−2^)		2	2	2	2
	Temperature (^o^C)		450	650	650	650
	Holding time (h)		3	1	2	3
Hydrogenation	Electrolyte		0.5 M Na_2_SO_4_	0.5 M Na_2_SO_4_	0.5 M Na_2_SO_4_	0.5 M Na_2_SO_4_
	Applied voltage (V)		5	5	5	5
	Processing time (s)		30	30	30	30

### Characterisations

The morphology of the prepared TNAs was investigated by field emission scanning electron microscopy (FESEM) (Tescan MIRA3 LMH) at 10 kV. The phase content was analysed by X-ray diffractometer (XRD) on a Rigaku Smart Lab SE diffractometer with patterns recorded in a range of 10~100°, Cu Kα, and the refinement of XRD patterns was performed using the software of Rigaku SmartLab Studio II. The detail information of morphology and crystal phase was acquired from transmission electron microscopy (TEM) (JEOL 2100 F) at 200 kV. The binding energy and chemical states were examined using X-ray photoelectron spectroscopy (XPS) (Escalab 250).

The electrochemical properties of the as-prepared H@TNA electrodes with the electroactive area of 4 cm^2^ were characterised by CHI660D electrochemical workstation. A typical three-electrode system with a 0.5-M Na_2_SO_4_ aqueous solution was employed, where H@TNAs, Pt sheet and saturated calomel electrode perform as a working electrode, counter electrode and reference electrode, respectively. The potential window of cyclic voltammetry (CV) and galvanostatic charge/discharge tests was − 0.3~0.5 V. The electrochemical impedance spectroscopy (EIS) measurement was performed in a frequency range of 0.1 Hz to 1 MHz with an AC signal amplitude of 10 mV without a bias potential.

## Results and Discussion

**Fig. 2:**
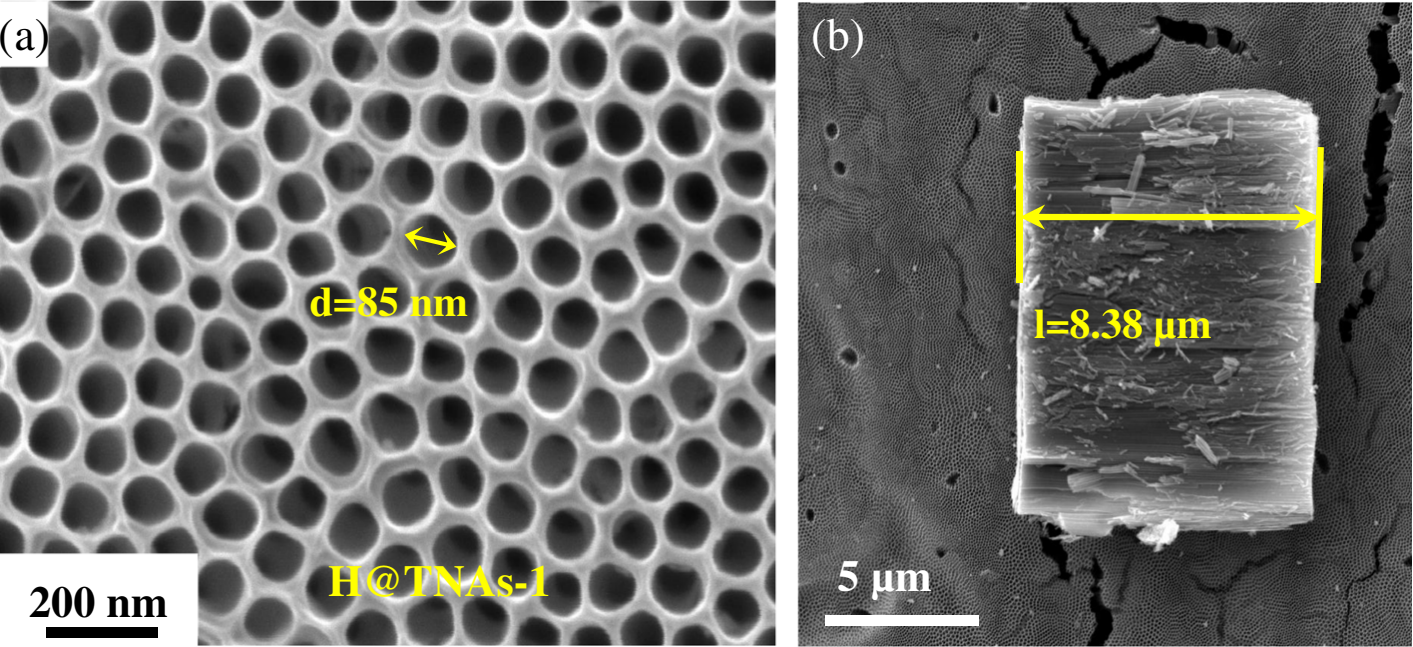
SEM images of H@TNAs-1. **a** The top view. **b** Cross-section of H@TNAs-1

The morphology of H@TNAs-1 is shown in Fig. 2. The H@TNAs-1 have a diameter of 85 ± 10 nm and a tube length of 8.3 ± 0.3 μm and maintain a relatively complete tubular structure even after a long period of high-temperature annealing.

**Fig. 3:**
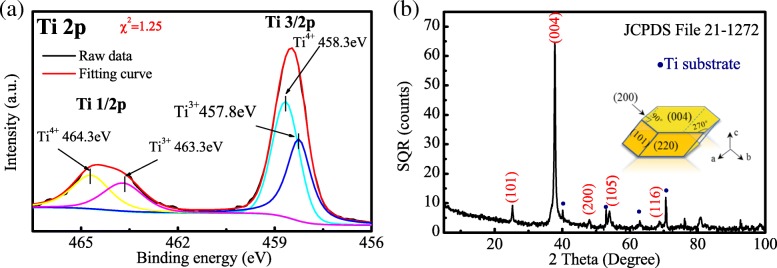
**a** Ti 2p XPS spectra of H@TNAs-1. **b** XRD pattern of H@TNAs-1 and sketch of the plate-like anatase grain with preferred growth

XPS was used to determine the chemical states of Ti and O. Peaks corresponding to typical Ti^4+^–O bonds can be observed at 458.3 eV for Ti^4+^ 2p_3/2_ and 464.3 eV for Ti^4+^ 2p_1/2_ in Fig. 3a. In addition, two peaks located at 457.8 eV and 463.5 eV can be assigned to Ti^3+^ 2p_3/2_ and Ti^3+^ 2p_1/2_, respectively, indicating the characteristic of a mixed-valence titanium system (Ti^4+^ and Ti^3+^).

Figure 3b shows the XRD pattern of H@TNAs-1. Almost all the diffraction peaks of H@TNAs-1 could be well indexed to anatase TiO_2_. It was worth to note that the abnormal extremely sharp peaks were assigned to anatase (004) planes, which indicated that the H@TNAs-1 may possess the crystal orientation of {001} facets. To determine the crystal growth of anatase, the texture refinement was performed by using whole powder pattern fitting (WPPF) method, based on March-Dollase function (1) (*W*(*α*)), ellipsoid model [47].

1 $$ W\left(\alpha \right)={\left({r}_n^2\mathrm{co}{\mathrm{s}}^2{\alpha}_{n,h}+{r}_n^{-1}\mathrm{si}{\mathrm{n}}^2{\alpha}_{n,h}\right)}^{-3/2} $$

where *α*_*n*, *h*_ represented the angle between the orientation vector and the diffraction plane vector. The coefficient of *r*_*n*_ reflected the preferred orientation strength. For *r*_*n*_ = 1, the growth of a grain was in random orientation; for *r*_*n*_ < 1, there is a preferred orientation by plate crystallites with the orientation vector perpendicular to the plate surface; and for *r*_*n*_ > 1, the grain grows preferentially by needle crystallites with the orientation vector parallel to the longitudinal direction of the needle [48, 49]. The parameters involved in the XRD refinement were listed in Additional file 1: Table S1, and the fitting results were shown in Additional file 1: Figure S1. The value of *r*_(004)_ for H@TNAs-1 was 0.2721. The results of refinement demonstrated that the anatase grains grew preferentially in <001> direction with plate crystallite which resulted in a high aspect ratio of {001} facets, seen in the inset of Fig. 3b.

**Fig. 4:**
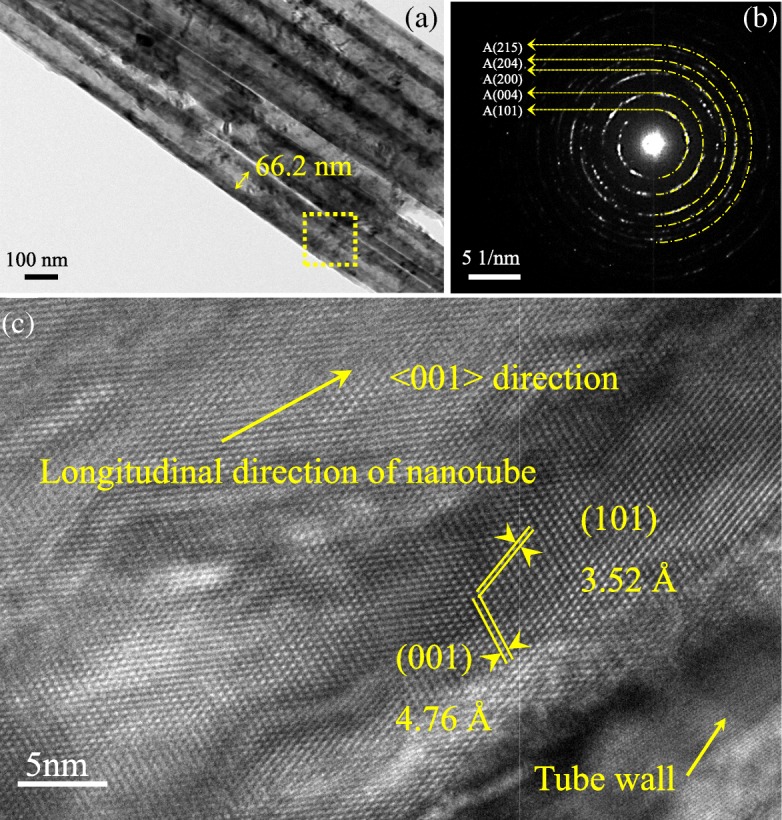
**a** TEM image of H@TNAs-1. **b** Corresponding selected area electron diffraction (SAED) patterns of the dotted area in a. **c** High-resolution TEM (HR-TEM) image of H@TNAs-1

To further investigate the detailed morphology and microstructure of H@TNAs-1, TEM, selected area electron diffraction (SAED) and HR-TEM images were used. Figure 4a displays a typical TEM image of H@TNAs-1. The inner diameter of H@TNAs-1 was ~ 66 nm. The SAED pattern of H@TNAs-1 in Fig. 4b depicted the diffraction rings, suggesting that the H@TNAs-1 presented in the form of polycrystals. Moreover, the surface of the H@TNAs-1 was found to become amorphous after hydrogenation, while the surface of untreated TNAs was highly crystalline, which was shown in Additional file 1: Figure S2. Such disorder structures were created by the hydrogenation, and this phenomenon was also reported in previous literatures [28, 50, 51]. Such disordered layers would provide an extra amount of carrier and promote the quick entry and exit of carriers during fast charge/discharge [52].

It was worth to note that, after performing the refinement of XRD patterns, anatase grains were found to grow preferentially along the {001} facets in a plate shape. And the lattice fringes assigned to anatase (001) planes arranged in a regular sequence and parallel to <001> direction were clearly observed, illustrated in Fig. 4c, demonstrating that the anatase crystallites stacked along the direction of tube length and perpendicular to the substrate. Such structure would favour the transfer of electrons along the <001> direction and elongate the electron diffusion lengths to several hundreds of micrometres [17, 53].

**Fig. 5:**
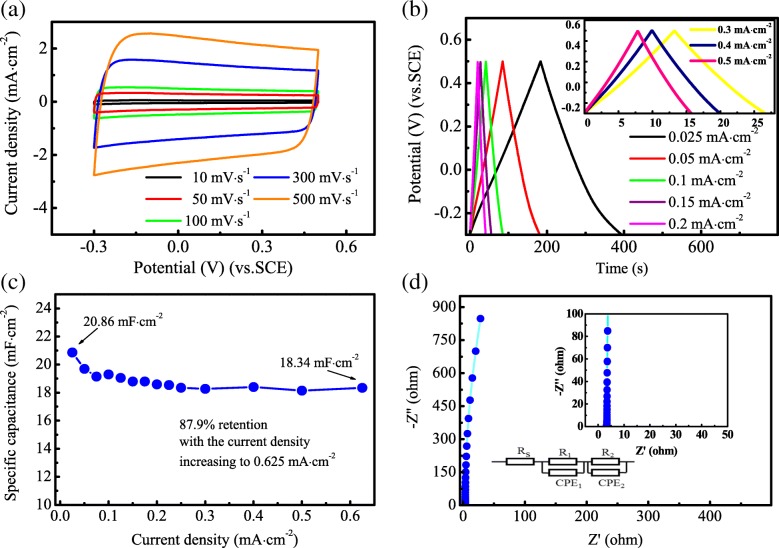
Supercapacitive performance of H@TNAs-1. **a** CV curves collected at various scan rates ranging from 10 to 500 mV s^−1^. **b** Galvanostatic charge/discharge curves at various current densities ranging from 0.025 to 0.5 mA cm^−2^, inset is the enlargement of the galvanostatic charge/discharge curves at higher current densities. **c** Nyquist plots collected at a frequency from 100 kHz to 10 mHz, with inset showing an enlargement of the high-frequency regions and a fitting equivalent circuit. **d** Specific capacitance of H@TNAs-1 measured as a function of current density

The electrochemical properties of H@TNAs-1 were evaluated firstly by cyclic voltammetry (CV), within a potential window of − 0.3~0.5 V (vs. SCE) at various scan rates from 10 to 500 mV s^−1^. As shown in Fig. 5a, the CV curves displayed ideal quasi-rectangular shapes even at the highest scan rate of 500 mV s^−1^, suggesting H@TNAs-1 exhibited an extraordinary capacitive property. The charge/discharge curves at various current densities were shown in Fig. 5b; the curves kept good linearity and symmetry regardless of the current density, indicating the excellent reversibility of charge/discharge process. The specific capacitance of H@TNAs-1 was calculated by Eq. (2) [54, 55]:

2 $$ C=\frac{2{i}_m\int Vdt}{{\left.{V}^2\right|}_{v_i}^{v_f}} $$

where *i*_*m*_ was the charge/discharge current density, ∫*Vdt* was the integral area surrounded by charge/discharge curve and *x* axis, *V*_*f*_ was the upper limit of potential window and *V*_*i*_ was the lower limit. H@TNAs-1 delivered a specific capacitance as high as 20.86 mF cm^−2^ at the current density of 0.025 mA cm^−2^ which was relatively higher than those random oriented H@TNAs reported in previous literature [19, 20, 28, 43] (summarised in Additional file 1: Table S2) and kept a retention of 87.9% with the current density increasing to 0.625 mA cm^−2^ as shown in Fig. 5c.

EIS measurement was performed to analyse the impedance behaviour of the electrochemical cells with the H@TNAs-1 as the working electrode. As shown in Fig. 5d, the Nyquist plots of H@TNAs-1 were nearly vertical to *Z*′ axis, and there was no noticeable semicircle in high-frequency region, indicating the approximately ideal capacitive behaviour and the superior conductivity of H@TNAs-1. To quantitatively investigate the impedance behaviour, an equivalent circuit, as shown in the inset of Fig. 5d, was used here to fit the Nyquist plots. *R*_s_ represented the series resistance mainly composed of the substrate and Na_2_SO_4_ aqueous solution, so the values of *R*_s_ were basically the same. A constant phase element CPE_1_ and *R*_1_ were used to fit the interfacial capacitor behaviour during the charge/discharge process, taking into account the deviation from ideal double layer structure on the electrode surface. The fitting parameters were listed in Additional file 1: Table S4 in detail. H@TNAs-1 delivered a relatively small diffusion resistance of 0.3039 Ω.

**Fig. 6:**
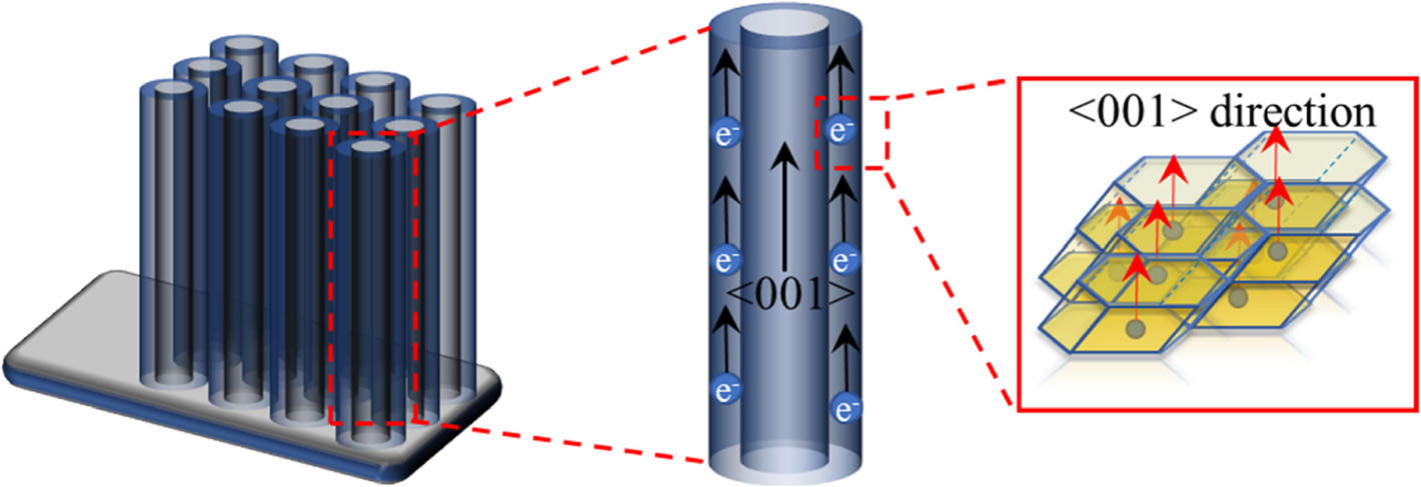
Schematic diagrams showing the efficient transfer of carrier along <001> direction within H@TNAs-1

The distinctive supercapacitive performances of H@TNAs-1 could attribute to the synergistic mechanisms as followed. The surface amorphous layers were created by the electrochemical hydrogenation. Related to the nature of amorphous structure, the homogeneous feature gave the amorphous material with isotropic ion diffusion and more percolation pathways, providing an open framework and more active sites and facilitating the fast electrode kinetics, which can favour the accumulation and intercalation/de-intercalation of electrolyte carriers on the surface of TNAs [52]. In addition, the process of hydrogenation can be understood as introducing oxygen vacancies (*V*_O_) in the TiO_2_ lattice. Then, oxygen deficiency transferred its extra two electrons to the adjacent two Ti^4+^ atoms to form Ti^3+^. So, there would be an additional free electron in the 3d orbital. Hence, the carrier concentration within TNAs was increased significantly. According to Boltzman theory, the conductivity was proportional to the carrier concentration [56, 57]. More importantly, the plate anatase crystallites stacked perpendicularly to the substrate along the <001> direction can provide an efficient highway for carrier transfer within H@TNAs-1 as shown in Fig. 6.

According to the results obtained above, apparently, the crystal structure has dramatic effects on the electrochemical performances of hydrogenated TiO_2_ nanotube arrays. Rutile/anatase interphase synergistic effect has been commonly used to improve the performance in photoelectrochemical and photocatalytic systems [58, 59], whether it can serve better supercapacitive properties for hydrogenated <001> oriented TNAs. To confirm this, the electrochemical performances of rutile/anatase <001> oriented TNAs were further investigated on the basis of the above-mentioned work.

As described in the experiment section, the rutile/anatase <001> oriented TNAs were fabricated by raising the annealing temperature to 650 °C then adjust the annealing time ranging from 1 to 3 h to obtain TNAs with different ratio of rutile/anatase. After the annealing treatment, the electrochemical hydrogenation was carried out in the same condition as H@TNAs-1 did.

**Fig. 7:**
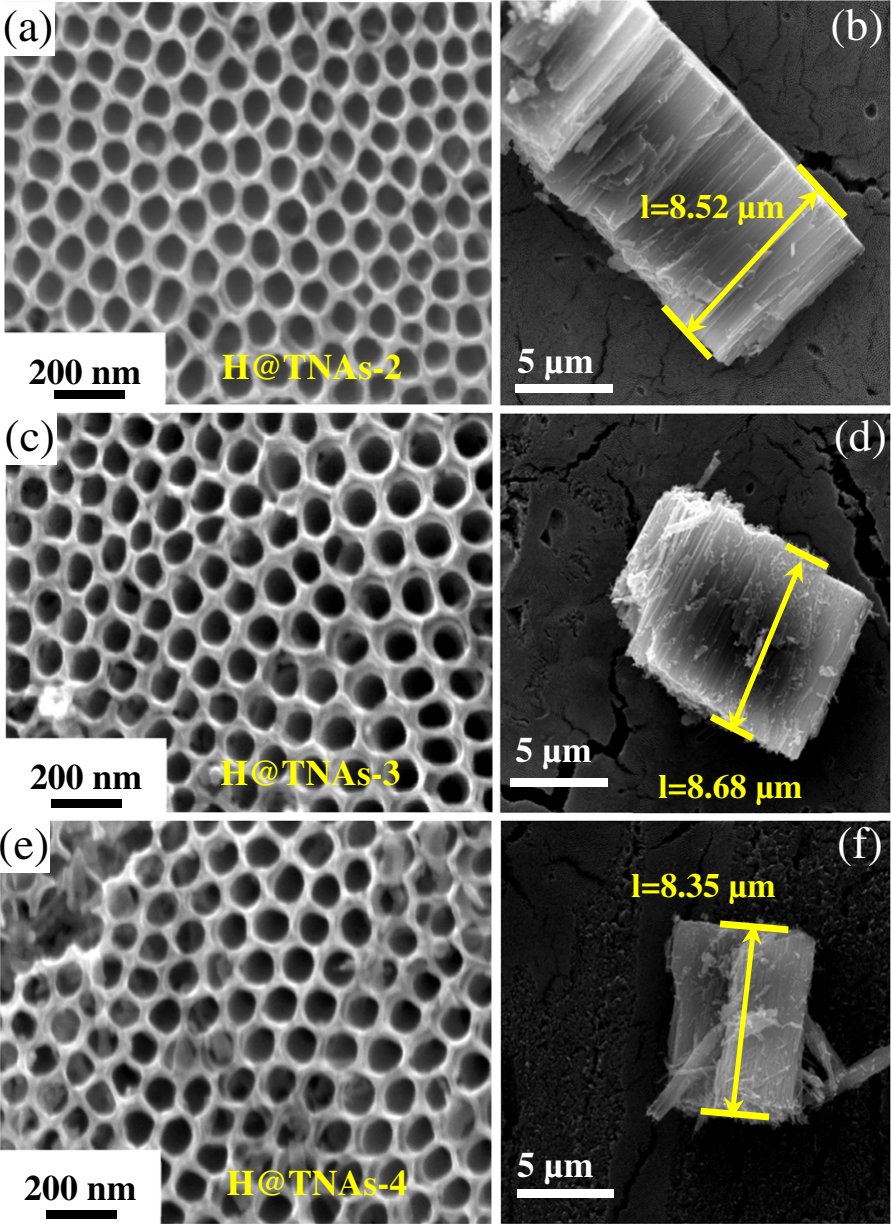
SEM images of **a** H@TNAs-2, **c** H@TNAs-3 and **e** H@TNAs-4. **b**, **d**, **f** The cross-sections of H@TNAs-2, H@TNAs-3 and H@TNAs-4, respectively. The as-prepared H@TNAs have a diameter of 85 ± 10 nm and a tube length of 8.5 ± 0.3 μm

The morphology of the electrodes has tremendous influences on its electrochemical properties, especially for supercapacitors. As shown in Fig. 7, the as-prepared H@TNAs-2, H@TNAs-3 and H@TNAs-4 were basically the same with H@TNAs-1 in topological dimension. Thus, the effect of morphology on the supercapacitor performances was eliminated.

**Fig. 8:**
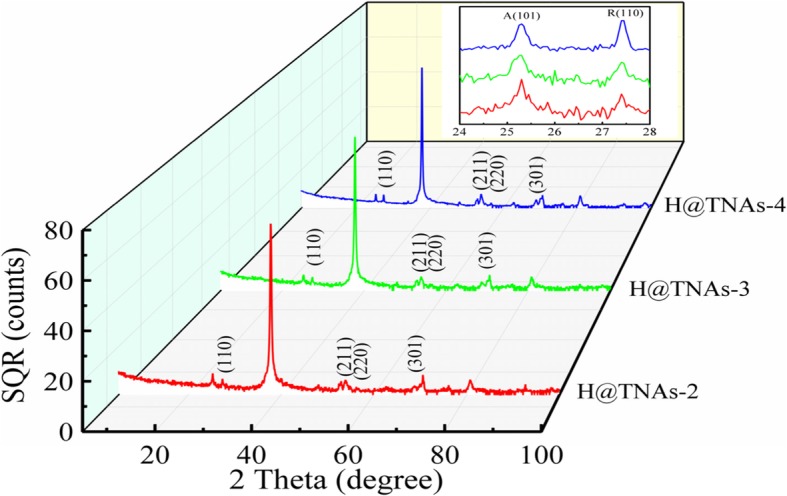
XRD patterns of H@TNAs-2, H@TNAs-3 and H@TNAs-4. Inset is the enlargement of the range from 24 to 28°

As shown in Fig. 8, with the annealing temperature raising to 650 °C, the characteristic peaks of rutile appeared in the XRD patterns of H@TNAs-2, H@TNAs-3 and H@TNAs-4 (JCPDS File 21-1276), centred at 27.45°, 54.32°, 56.6° and 69.0° which were corresponding to rutile (110), (211), (220) and (301) planes, respectively, suggesting that the transformation from anatase to rutile was activated when annealing at 650 °C. And with the extension of holding time, the intensity of peak assigned to rutile (110) plane increased gradually, demonstrating the increase in rutile content. Furthermore, H@TNAs-2, H@TNAs-3 and H@TNAs-4 also possessed the <001> texture which was determined in the framework of WPPF. As shown in Additional file 1: Figure S1 and Table S1, anatase grains still possessed the preferential growth along the <001> direction with a plate shape when the annealing temperature was 650 °C.

**Fig. 9:**
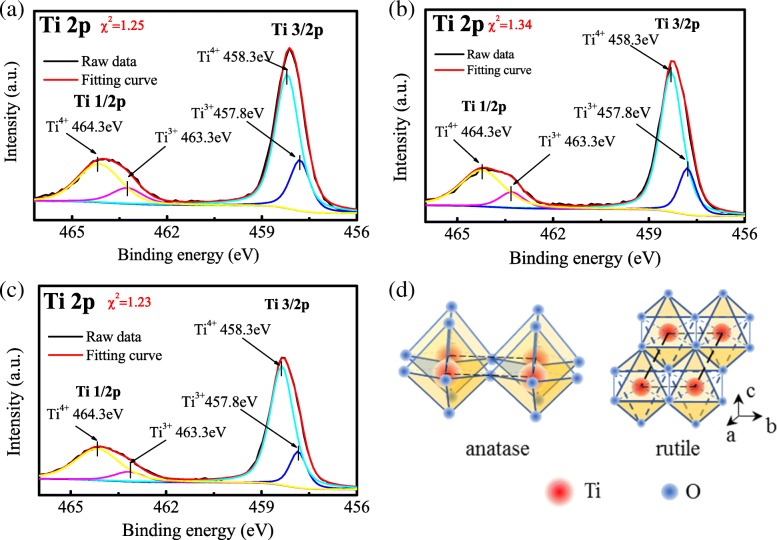
XPS spectra of **a** H@TNAs-2, **b** H@TNAs-3 and **c** H@TNAs-4. **d** Sketches of the crystal structure of anatase and rutile [37, 38]

The peaks at 458.5 eV for Ti^4+^ 2p 3/2, 457.8 eV for Ti^3+^ 2p 3/2, 464.3 eV for Ti^4+^ 2p 1/2 and 463.3 eV for Ti^3+^ 2p 1/2 in the Ti 2p XPS spectra suggested the coexistence of the Ti^4+^ and Ti^3+^. Moreover, with the rutile content increasing, there was a gradual reduction in relative concentration of Ti^3+^. The decline of Ti^3+^ concentration maybe caused by the crystal structural difference of anatase and rutile. As shown in Fig. 9d, anatase is composed of [TiO_6_] octahedrons with the corner-shared structure, while rutile has [TiO_6_] octahedra joined by sharing the octahedral edges, which is more stable than the corner-shared structure [60, 61]. Therefore, it was more difficult to create defects in rutile. In other words, less oxygen vacancies (*V*_O_s) generated during the hydrogenation process.

A straightforward method [62] was employed to evaluate the relative concentration of Ti^3+^ based on the ratio of two peak areas of Ti^3+^ and Ti^4+^:

3 $$ \%\mathrm{T}{\mathrm{i}}^{3+}=\left[\frac{A_{{\mathrm{Ti}}^{3+}}}{A_{{\mathrm{Ti}}^{3+}}+{A}_{{\mathrm{Ti}}^{4+}}}\right]\times 100\% $$

where %Ti^3+^ represented the relative concentration of Ti^3+^ in each sample, and *A*_Ti_^3+^ and *A*_Ti_^4+^ were the total areas of the peaks attributed to Ti^3+^ and Ti^4+^, respectively, in XPS spectra. Relative concentrations of Ti^3+^ of each sample were listed in Table 3.

**Table 3:** Relative concentrations of Ti^3+^ of each sample

Samples	H@TNAs-2	H@TNAs-3	H@TNAs-4
Relative concentration of Ti^3+^ (%)	30.9	21.29	11.8

**Fig. 10:**
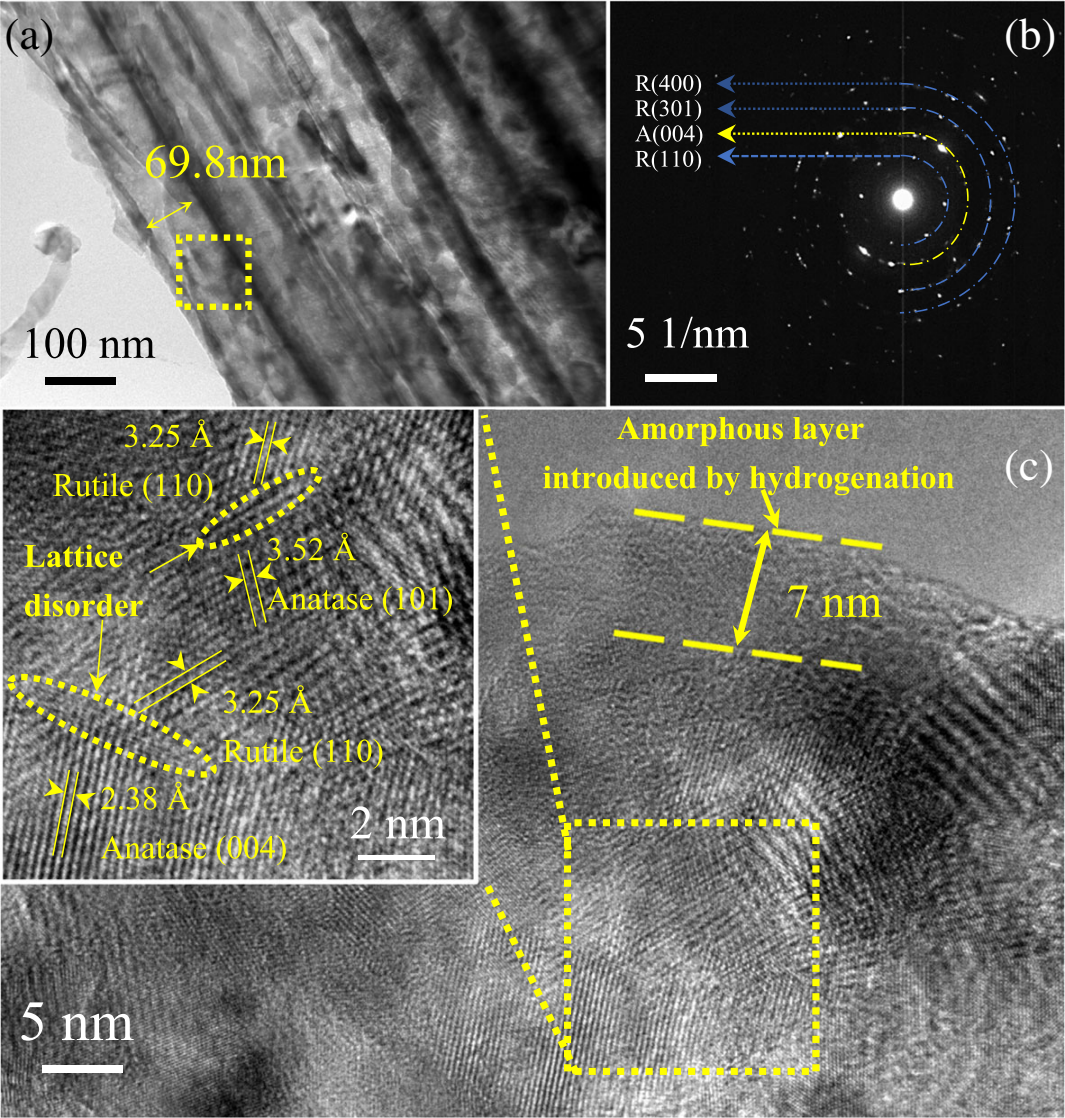
**a** TEM image of H@TNAs-2. **b** Corresponding SAED patterns of the dotted area in a. HR-TEM images of **c** H@TNAs-3. The inner diameters of all samples are ~ 70 nm, regardless of the annealing temperature

Figure 10 and Additional file 1: Figure S3 show the TEM images of H@TNA-2, H@TNAs-3 and H@TNAs-4. Figure 10a and Additional file 1: Figure S3(a) S3(b) reveal that all the samples maintain a complete tubular structure which was basically the same with that of H@TNAs-1. As shown in Fig. 10b, the SAED patterns of H@TNAs-2 depicted diffraction rings, suggesting that as-prepared H@TNAs annealed at 650 °C also presented in the form of polycrystals. The amorphous layer induced by hydrogenation became thinner with the content of rutile increasing due to a more stable surface crystal structure. As shown in Fig. 10c and Additional file 1: Figure S3(c), the thickness of the hydrogenated amorphous layer for H@TNAs-3 was approximately 7 nm while that for H@TNAs-4 was only about 1 nm. Furthermore, the layers of lattice disorder with the thickness of only several nanometres can be seen between the anatase and rutile grains, the dotted area of the inset of Fig. 10c, Additional file 1: Figure S3(c) and S3(d). According to the mechanism of the transformation from anatase to rutile, the process of anatase converting to rutile was not instantaneous but time-dependent, and the transition rate would become slower with the process going on [63, 64]. This was a nucleation and growth process. Rutile may nucleate at the surface of anatase grain first, then the phase transition interface moves forward to the interior of the anatase phase. Since the breaking and reforming of the Ti–O bonds were involved in the phase transition, the presence of lattice disorder layer between two phases was inevitable. That means, the Ti–O bonds assigned to anatase broke to form a disordered layer firstly, then the [TiO_6_] basic units rearranged into rutile phase [65, 66]. And the disordered layer became thinner with rutilisation proceed. When the annealing time was 3 h, the lattice disorder layers were too thin to be detected in the HR-TEM image. On the one hand, these disordered structures can provide a small amount of carrier to improve interfacial capacitance and promote the quick entry and exit of carriers within grains [52] just as discussed in the previous section. On the other hand, the massive of lattice disorders would lead to the significant rise in impedance, because the carrier transport would be inevitably affected by disorder scattering within disordered structures which may make the increase in electron-hole recombination rate. Furthermore, the rutile phase linking the adjacent anatase phase acted as a ‘bridge’ when the annealing time is more than 2 h. Due to the lower electron affinity of rutile, such rutile ‘bridges’ would facilitate the carrier transfer [67, 68].

**Fig. 11:**
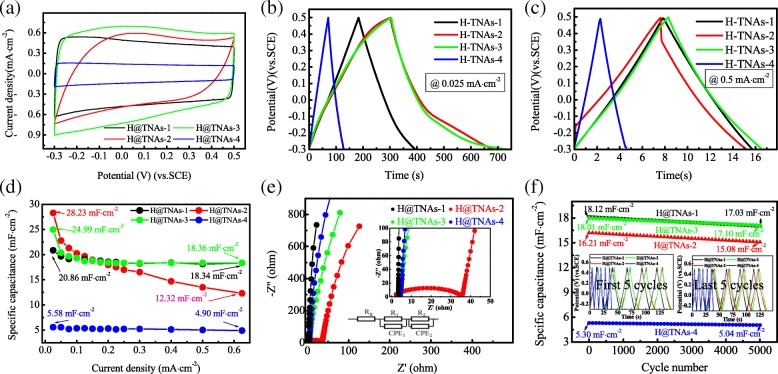
Supercapacitive properties of oriented H@TNAs with mixed crystal structures. **a** CV curves collected at the scan rate of 100 mV s^−1^. Galvanostatic charge/discharge curves at current densities of **b** 0.025 and **c** 0.5 mA cm^−2^. **d** Specific capacitance of as-prepared H@TNAs measured as a function of current density. **e** Nyquist plots of as-prepared H@TNAs. **f** Cyclic performance of as-prepared H@TNAs, insets are the galvanostatic charge/discharge curves of the first 5 cycles and the last 5 cycles

Figure 11a shows the CV curves of as-prepared H@TNAs, which exhibited quasi-rectangular shapes except that of H@TNAs-2. The distortion of the CV curves of H@TNAs-2 can be attributed to the large polarisation at high scan rates, indicating the larger intrinsic resistance of H@TNAs-2. Such phenomenon indicates that the resistance of H@TNAs decreased with the improvement in rutile content. Yet, the current densities of the CV curves for H@TNAs-4 were much smaller than those of H@TNAs-2 and H@TNAs-3 suggesting the limited charge storage capability of H@TNAs-4.

Figure 11b and c display the galvanostatic charge/discharge curves of as-prepared H@TNAs. The charge/discharge curves of all the samples mentioned above were linear with quasi-symmetric triangular shapes at high current densities (Fig. 11c). While at small current densities, there were slight slope variations on the discharge curve at − 0.1 V for both H@TNAs-2 and H@TNAs-3, but the inflexion points disappeared when the current density raised to 0.5 mA cm^−2^, which can be regarded as the impedance of lattice disorders within the H@TNAs. At larger current density, the driving force was big enough to make carriers pass through the layer of lattice disorder directionally and quickly, so there was no inflexion point at − 0.1 V when the charge/discharge current was high. And for H@TNAs-1 and H@TNAs-4, which contained only tiny amounts of lattice disordered structures within nanotube arrays, the charge/discharge curves of H@TNAs-4 kept the linear shapes. The specific capacitances of H@TNAs-1, H@TNAs-2, H@TNAs-3 and H@TNAs-4 as a function of current density were compared in Fig. 11d. Based on the charge/discharge curves obtained, using Eq. (2), the galvanostatic charge/discharge-specific capacitances of H@TNAs-2, H@TNAs-3 and H@TNAs-4 were calculated. As shown in Additional file 1: Table S2, it was evident that the capacitances achieved in this work were much higher than that of the relevant previous reports [19, 20, 28, 43] taking the length of tube into account. H@TNAs-3 showed a relatively higher specific capacitance of 24.99 mF cm^−2^ at the current density of 0.025 mA cm^−2^, more than 73% capacitance can be retained at such a high current density of 0.625 mA cm^−2^, demonstrating excellent rate capability. Although H@TNAs-2 shows a much larger specific capacitance compared to other electrodes as high as 28.23 mF cm^−2^ at the current density of 0.025 mA cm^−2^, the capacitance of H@TNAs-2 declined quickly to 13.55 mF cm^−2^ when the current density increased to 0.625 mA cm^−2^. Despite the low specific capacitance, H@TNAs-4 also exhibited strikingly outstanding rate performance with only 12% capacitance loss at high current densities. In addition, H@TNAs-2 showed a large IR drop suggesting the large intrinsic resistance as listed in Additional file 1: Table S3.

The behaviour of galvanostatic charge/discharge was bound up with the impedance properties. Electrochemical impedance spectroscopy (EIS) was carried out to further understand the electrochemical behaviour of as-prepared H@TNAs. In order to determine the effect of rutile content on the electrochemical performance of the electrodes, the impedance spectra of H@TNAs-1 was also involved. As shown in Fig. 11e, the Nyquist plots of H@TNAs-3 and H@TNAs-4 also exhibited nearly vertical lines to *Z*′ axis, just bent slightly down to the *Z*′ axis compared with those of H@TNAs-1, indicating the slight increase in resistance both of H@TNAs-3 and H@TNAs-4. But for H@TNAs-2, there was a flattened semicircle in the high-frequency region, which suggested the much larger intrinsic resistance of H@TNAs-2 [69–71]. The equivalent circuit shown in the inset of Fig. 11e was used, to fit the Nyquist plots. Fitting parameters of oriented mix-crystalline H@TNAs were listed in Additional file 1: Table S4 in detail, in which those of H@TNAs-1 were involved. With the appearance of rutile, the carrier diffusion resistance *R*_2_ improved greatly from 0.30 to 29.28 Ω, then decreased to 1.16 Ω gradually with the prolongation of annealing time at 650 °C.

The cycling stability was one of the most important properties of supercapacitors; the as-prepared H@TNAs (H@TNAs-1, H@TNAs-2, H@TNAs-3 and H@TNAs-4) were subjected to a continuous cycling for 5000 cycles in the three-electrode configuration at the current density of 0.3 mA cm^−2^ within the potential window from − 0.3 to 0.5 V as shown in Fig. 11f. All the samples delivered excellent cycling stability. The retention rates of the specific areal capacitance were 94% for H@TNAs-1, 93% for H@TNAs-2, 95% for H@TNAs-3 and 95% for H@TNAs-4. The results were summarised in Additional file 1: Table S6. Additionally, the energy density and the power density of each sample were calculated at 0.3 mA cm^−2^ which were shown in Additional file 1: Table S7 in detail.

**Fig. 12:**
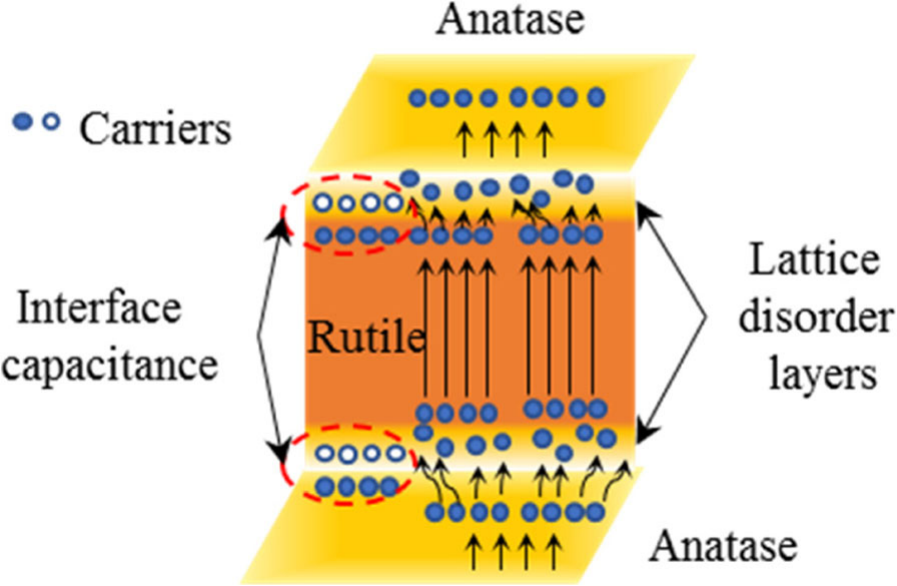
Sketch of the carrier transfer within H@TNAs with mixed crystal structures

Such results could be ascribed to the comprehensive effects of lattice disorder layer and rutile. When the annealing time was 1 h, the massive disordered structure can endow interface capacitance and small amounts of additional carrier but exacerbate the carrier inelastic scattering and electron-hole recombination resulting in a significant increase in impedance at the same time. As the annealing process went on, the rutile grain grew steadily, then connected with each other to form structures like ‘bridges’ linking the adjacent anatase grains. Since the electron affinity of rutile is lower than that of anatase, the ‘rutile bridge’ can promote the charge separation and transportation, resulting in an enhancement in carrier transmission efficiency [59, 67, 68]; hence, the drawbacks brought by the lattice disordered structures can be circumvent effectively. Figure 12 illustrated the carrier transfer within H@TNAs with mixed crystal structures. But a longer annealing duration would lead to a dramatic decline in capacitance, which could be ascribed to increased surface stability and the corresponding decrease in surface amorphous structures and carrier density.

## Conclusion

In this paper, highly ordered <001> oriented TiO_2_ nanotube arrays with different crystal structures have been fabricated via two-step anodisation and subsequent annealing in an argon atmosphere. After a facile electrochemical hydrogenation process, high-performance H@TNA electrodes were successfully synthesised. Combined with various characterisation, the effect of crystal structure on the supercapacitive performance of H@TNAs was elaborated. The results revealed that the supercapacitive performances could be enhanced remarkably by constructing proper crystal structure. Those H@TNAs with <001> orientation and rutile/anatase mixed crystal structure showed a significant enhancement in specific capacitance compared with random oriented anatase counterparts. At the annealing condition of temperature 450 °C and holding time 1 h, pure anatase TNAs with <001> orientation were obtained. After hydrogenation process, H@TNAs-1 exhibited a high specific capacitance of 20.86 mF cm^−2^. Such good performance can attribute to the comprehensive effect of hydrogenation process and < 001> orientation. The surface amorphous layers introduced by the hydrogenation process provided more electrochemical active sites and favoured the fast accumulation and intercalation/de-intercalation of electrolyte carriers on the surface of TNAs. Then, the structure of <001> direction preferential growth with plate crystallite stacking vertically to the substrate confined an efficient transfer highway for the large amounts of carriers introduced by hydrogenation process. When the annealing temperature rose up to 650 °C, the orientation of the nanotubes retained and the crystal transformation from anatase to rutile was activated. <001> oriented TNAs with different rutile/anatase ratios were synthesised by prolonging the annealing holding time. The specific capacitance of <001> oriented H@TNAs can be further improved by partial rutile/anatase transformation. The H@TNAs-3 sample, annealed at 650 °C for 2 h under Ar atmosphere before hydrogenation, delivered a relatively high specific capacitance of 24.99 mF cm^−2^, as well as an outstanding rate capability and good cyclic stability. The <001> orientation of anatase grains and the comprehensive effects of lattice disorder layers and rutile played important roles in the remarkable enhancement in supercapacitive properties of H@TNAs-3. Such findings would hold significant promise to provide new fundamental information for the design and fabrication of high-performance H@TNA heterostructures in energy storage fields.

## Additional File

Additional file 1: **Figure S1.** The measured and simulated XRD patterns of as-prepared H@TNAs. **Figure S2.** HR-TEM image of (a) H@TNAs-1 and (b) TNAs-1. **Figure S3.** Typical TEM image of (a) H@TNAs-3 and (b) H@TNAs-4 and HR-TEM image of (c) H@TNAs-2 and (d) H@TNAs-4. **Figure S4.** CV curves collected at different scan rates ranging from 10 to 500 mV s^−1^: (a) H@TNAs-2, (b) H@TNAs-3 and (c) H@TNAs-4. Galvanostatic charge/discharge curves at various current densities ranging from 0.025 to 0.5 mA cm^−2^, inset is the enlargement of the galvanostatic charge/discharge curves at higher current densities: (d) H@TNAs-2, (e) H@TNAs-3 and (f) H@TNAs-4. **Figure S5.** Surface morphology of each sample after 5000 cycles: (a) H@TNAs-1, (b) H@TNAs-2, (c) H@TNAs-3 and (d) H@TNAs-4. **Table S1.** The fitted March coefficient of the preferred orientation degree in <001> preferred plate anatase crystallite within the framework of March-Dollase function. **Table S2.** Comparison of the results of some oxygen-deficient TNAs with random orientation in the previous literature. **Table S3.** Equivalent series resistance of as-prepared H@TNAs. **Table S4.** Fitting parameters of the equivalent circuit for the Nyquist plots. **Table S5.** The calculations of C. **Table S6.** Comparison of the discharge-specific areal capacitances before and after 5000 cycles. **Table S7.** Energy densities and power densities of as-prepared H@TNAs. (DOCX 77429 kb)

## Data Availability

All data included in this study are available upon reasonable requests by contacting the corresponding author.

## Abbreviations

CV: Cyclic voltammetry
EIS: Electrochemical impedance spectroscopy
FESEM: Field emission scanning electron microscopy
H@TNAs: Hydrogenated TiO_2_ nanotube arrays
H@TNAs-1: Hydrogenated TiO_2_ nanotube arrays which was annealed at 450 °C for 3 h before hydrogenation process
H@TNAs-2: Hydrogenated TiO_2_ nanotube arrays which was annealed at 650 °C for 1 h before hydrogenation process
H@TNAs-3: Hydrogenated TiO_2_ nanotube arrays which was annealed at 650 °C for 2 h before hydrogenation process
H@TNAs-4: Hydrogenated TiO_2_ nanotube arrays which was annealed at 650 °C for 3 h before hydrogenation process
HR-TEM: High-resolution transmission electron microscopy
SAED: Selected area electron diffraction
TEM: Transmission electron microscopy
TNAs: TiO_2_ nanotube arrays
WPPF: Whole powder pattern fitting
XPS: X-ray photoelectron spectroscopy
XRD: X-ray diffractometer
