# Supplementary material for: An Important Factor Affecting the Supercapacitive Properties of Hydrogenated TiO2 Nanotube Arrays: Crystal Structure
Source: Nanoscale Res Lett. 2019 Jul 10;14:229. doi: 10.1186/s11671-019-3047-2 (PMC6620217; doi:10.1186/s11671-019-3047-2)
Supplement: Supplementary file 1 — Figure S1. The measured and simulated XRD patterns of as-prepared H@TNAs. Figure S2. HR-TEM image of (a) H@TNAs-1 and (b) TNAs-1. Figure S3. Typical TEM image of (a) H@TNAs-3 and (b) H@TNAs-4 and HR-TEM image of (c) H@TNAs-2 and (d) H@TNAs-4. Figure S4. CV curves collected at different scan rates ranging from 10 to 500 mV s−1: (a) H@TNAs-2, (b) H@TNAs-3 and (c) H@TNAs-4. Galvanostatic charge/discharge curves at various current densities ranging from 0.025 to 0.5 mA cm−2, inset is the enlargement of the galvanostatic charge/discharge curves at higher current densities: (d) H@TNAs-2, (e) H@TNAs-3 and (f) H@TNAs-4. Figure S5. Surface morphology of each sample after 5000 cycles: (a) H@TNAs-1, (b) H@TNAs-2, (c) H@TNAs-3 and (d) H@TNAs-4. Table S1. The fitted March coefficient of the preferred orientation degree in <001> preferred plate anatase crystallite within the framework of March-Dollase function. Table S2. Comparison of the results of some oxygen-deficient TNAs with random orientation in the previous literature. Table S3. Equivalent series resistance of as-prepared H@TNAs. Table S4. Fitting parameters of the equivalent circuit for the Nyquist plots. Table S5. The calculations of C. Table S6. Comparison of the discharge-specific areal capacitances before and after 5000 cycles. Table S7. Energy densities and power densities of as-prepared H@TNAs. (DOCX 77429 kb) [file 11671_2019_3047_MOESM1_ESM.docx]

**Supporting information**

**An important factor affecting the supercapacitive properties of hydrogenated TiO_2_ nanotube arrays: Crystal structure**

Wenyi Li ^1^, Wanggang Zhang ^1^, Taotao Li ^1^, Aili Wei ^1^,

Yiming Liu ^1,2 *^, Hongxia Wang ^1,*^

1 College of Materials Science and Engineering, Taiyuan University of Technology, Taiyuan Shanxi 030024, China

2 Shanxi Academy of Analytical Sciences, Taiyuan 030006, China

*Correspondence: [liuym812@163.com](mailto:liuym812@163.com) (M.L.); [wanghxia1217@163.com](mailto:wanghxia1217@163.com) (H.W.)

Email address: [liwenyi1126@126.com](mailto:liwenyi1126@126.com) (W.L.); [zwgang0117@163.com](mailto:zwgang0117@163.com) (W.Z); [xueyanles10@126.com](mailto:xueyanles10@126.com) (T.L); [slowly0535@126.com](mailto:slowly0535@126.com) (A.W.);

*Results of XRD refinement*

**

**

**Figure. S1** The measured and simulated XRD patterns of as-prepared H@TNAs

The texture refinement of anatase phase was performed, and the simulated results were consistent with measured data.

**Table. S1** The fitted March coefficient of the preferred orientation degree in <001> preferred plate anatase crystallite within the framework of March-Dollase function.

| Samples | r_(004)_ | χ^2^ |
| --- | --- | --- |
| H@TNAs-1 | 0.2721 | 1.2834 |
| H@TNAs-2 | 0.2540 | 1.2286 |
| H@TNAs-3 | 0.2491 | 1.2443 |
| H@TNAs-4 | 0.2462 | 1.2655 |

*TEM images of as-prepared H@TNAs*


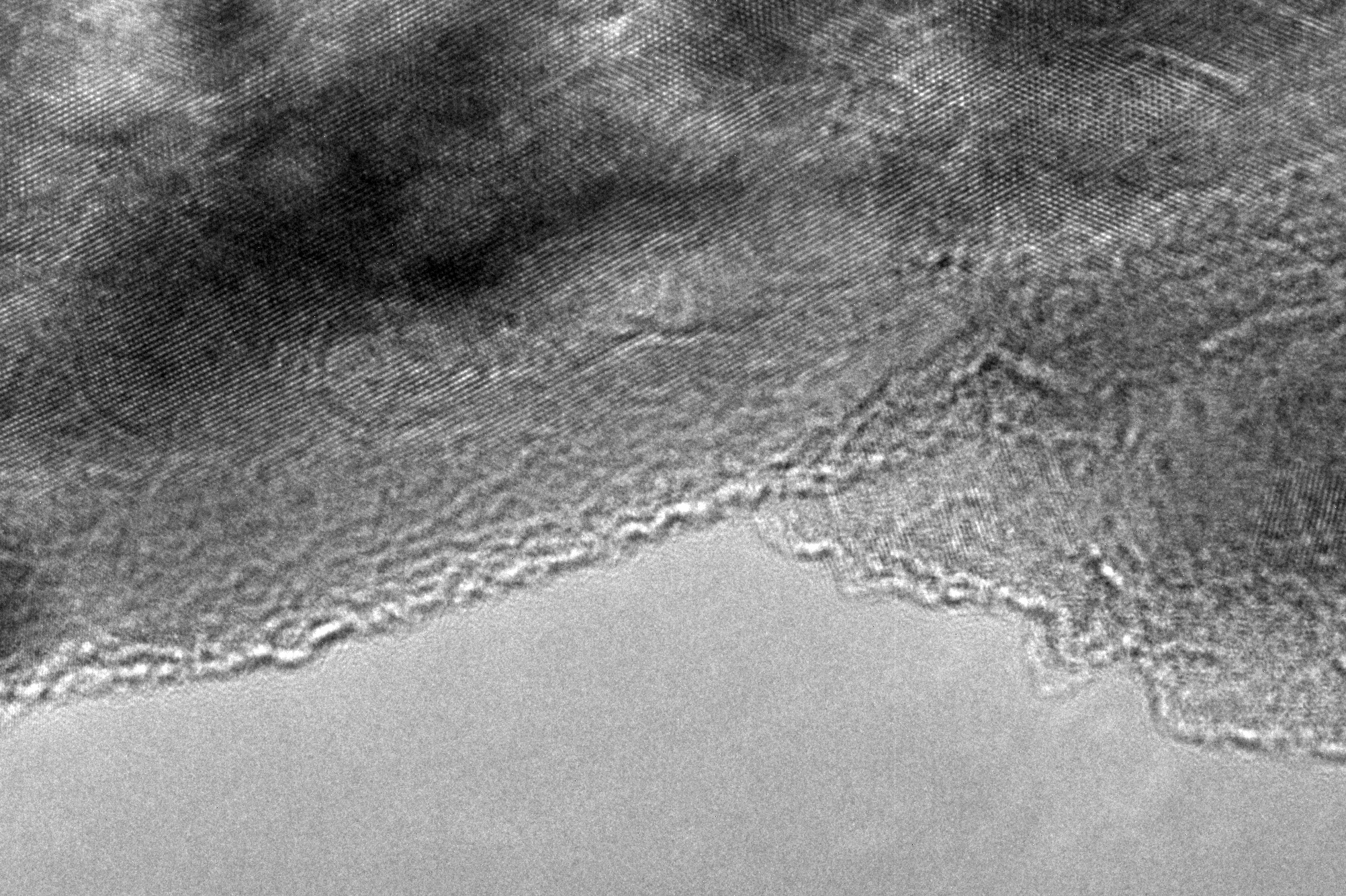


3.52 Å

(101)

**Amorphous layer**

**introduced by hydrogenation**

4.76 Å (001)

10 nm


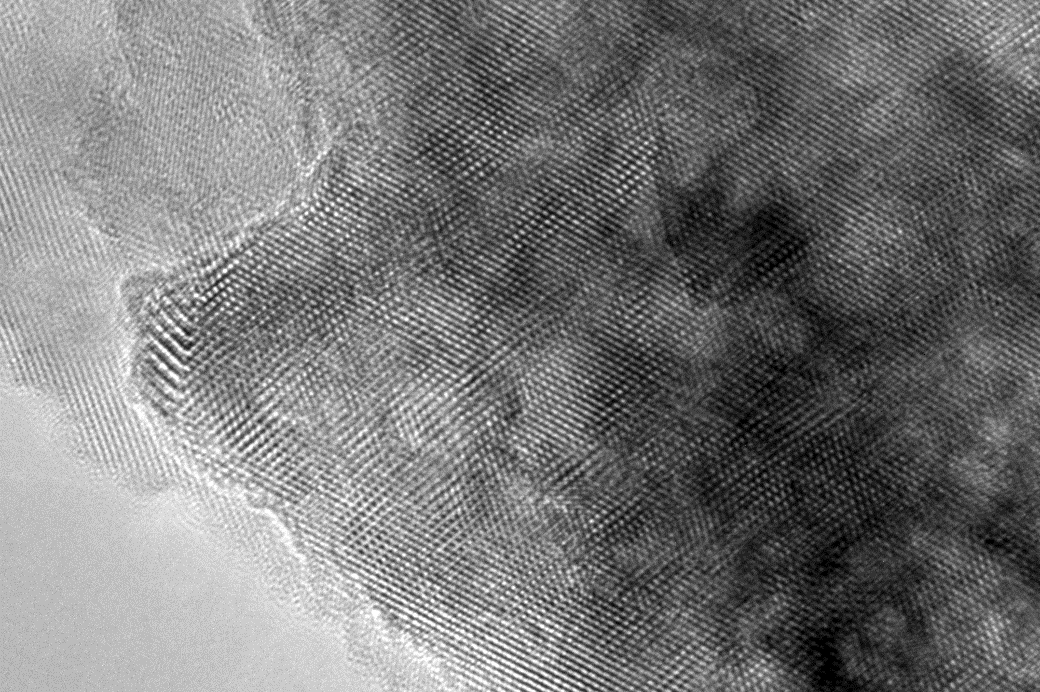


5nm

3.52 Å

(101)

4.76 Å

(004)

(b)

**Highly crystallized boundary**

(a)

12 nm

**Figure. S2** HR-TEM image of (a)H@TNAs-1 and (b)TNAs-1.


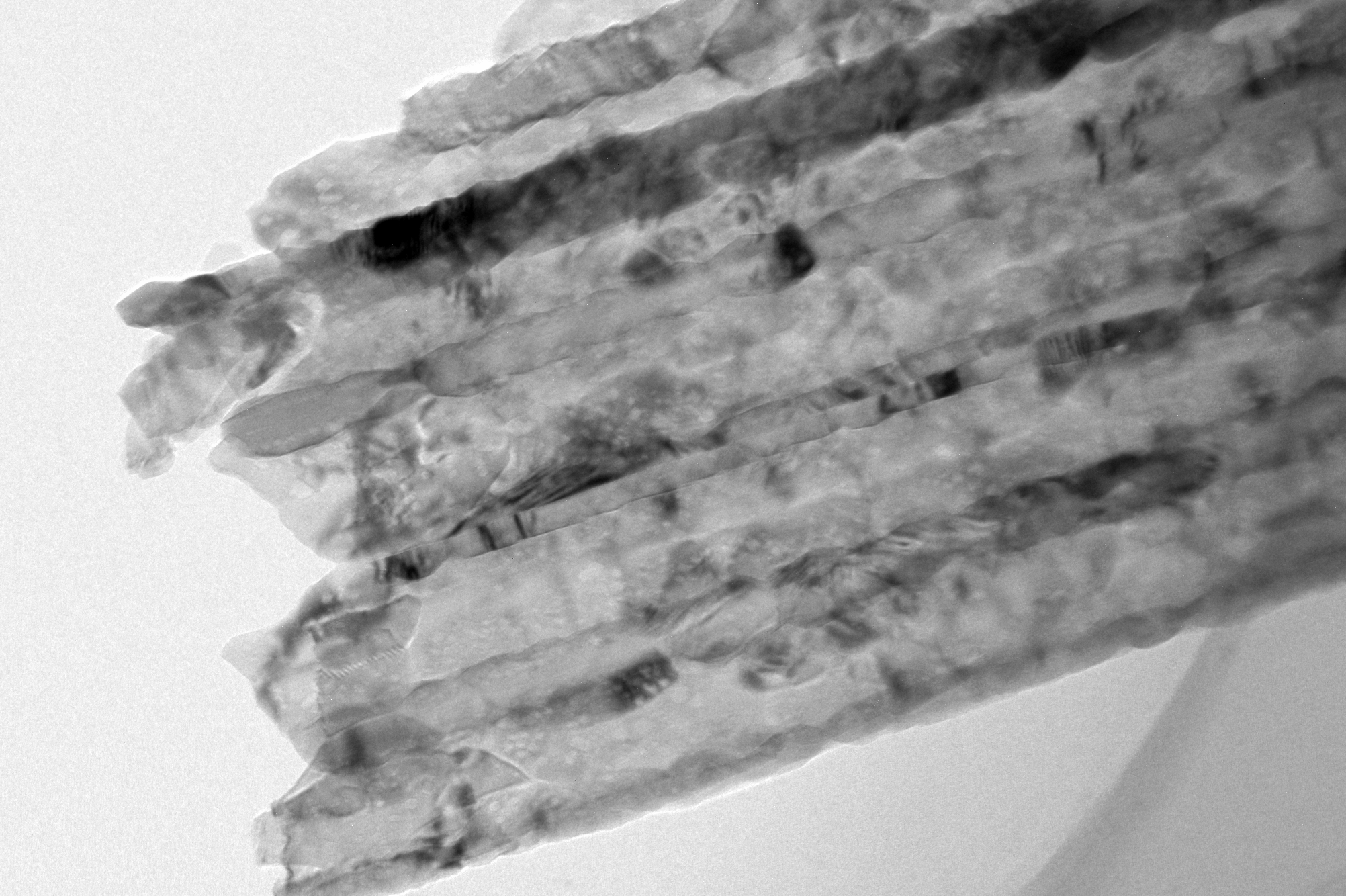


100 nm

(a)


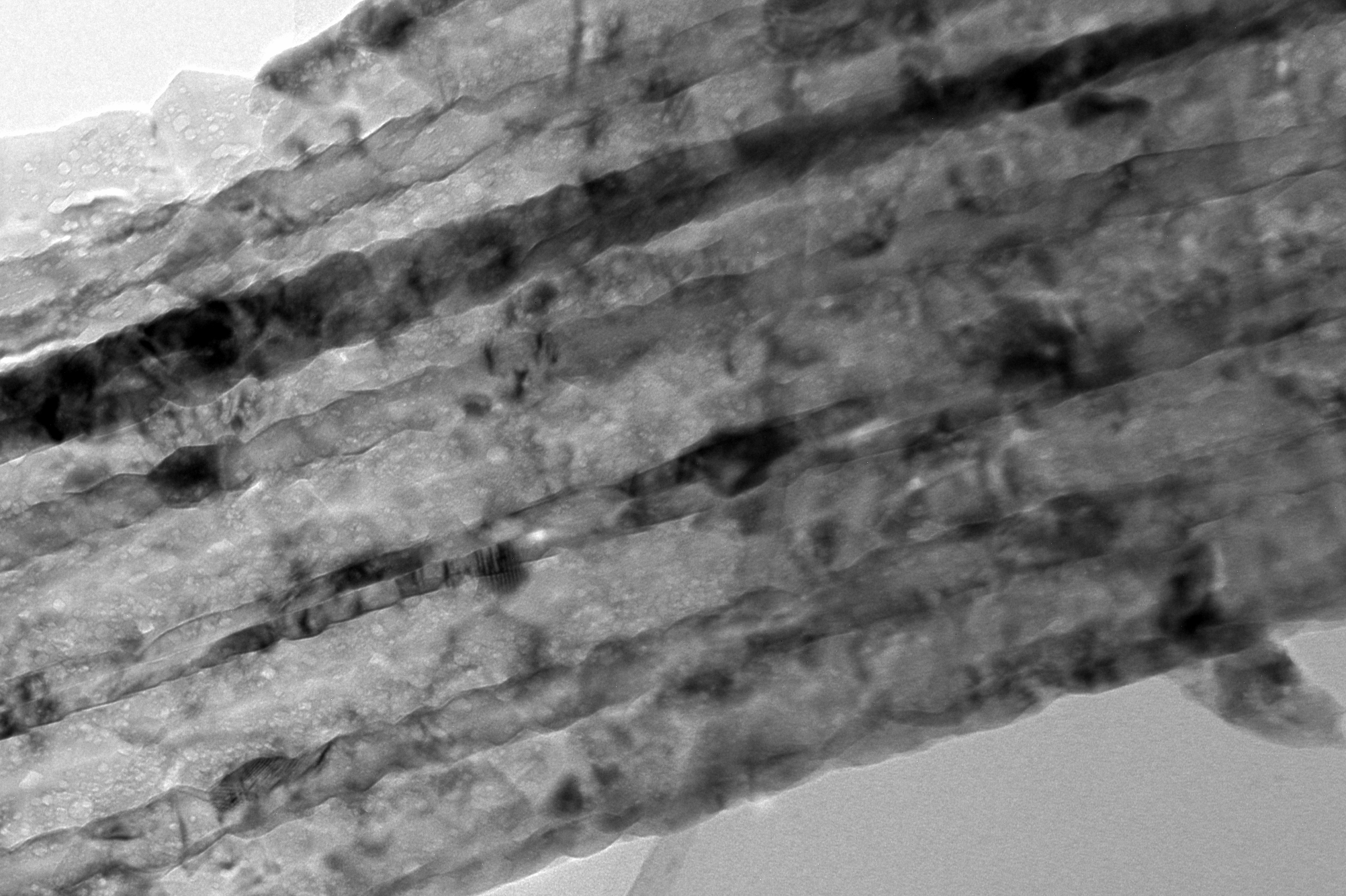


100 nm

(b)


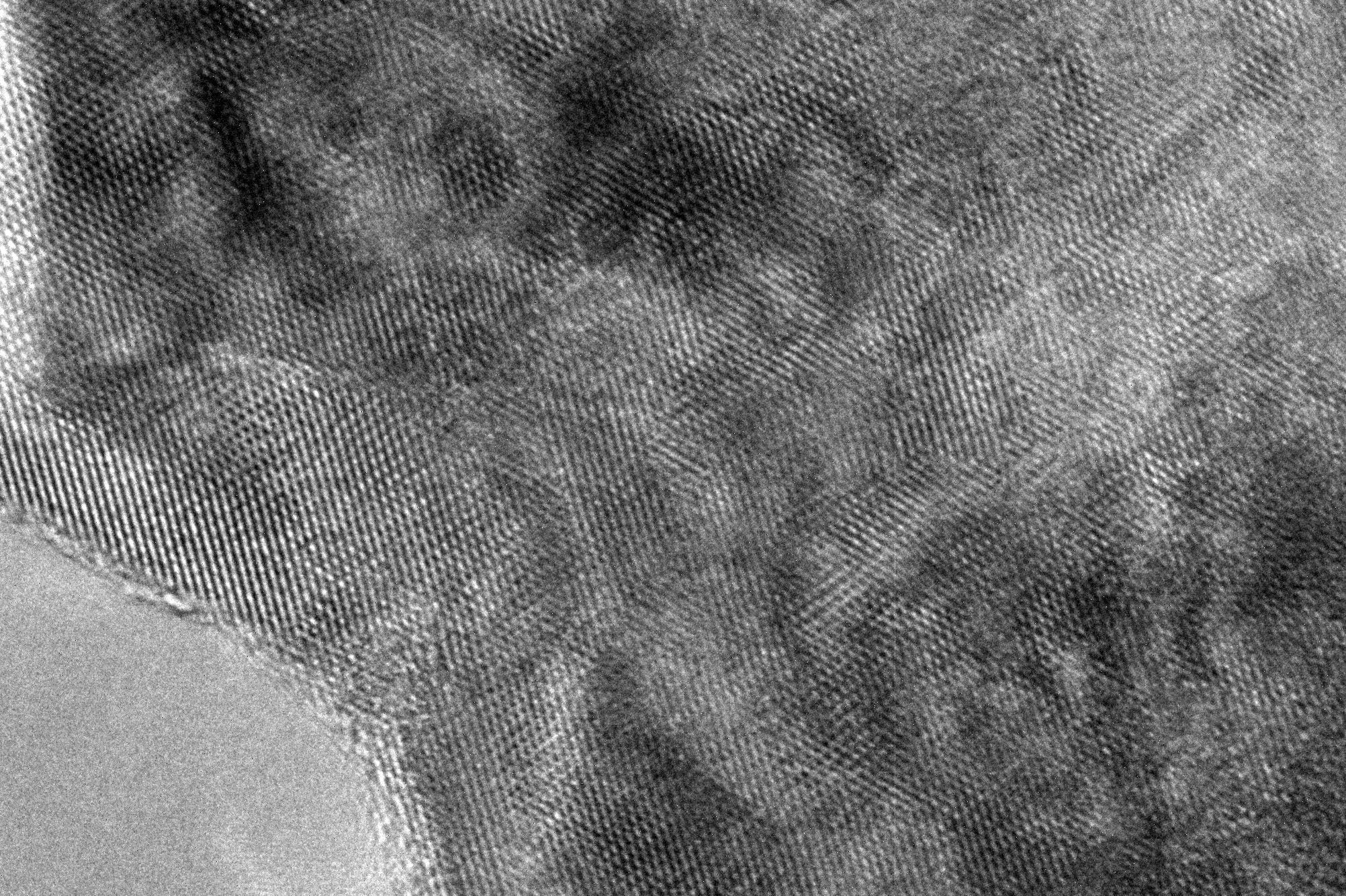


5 nm


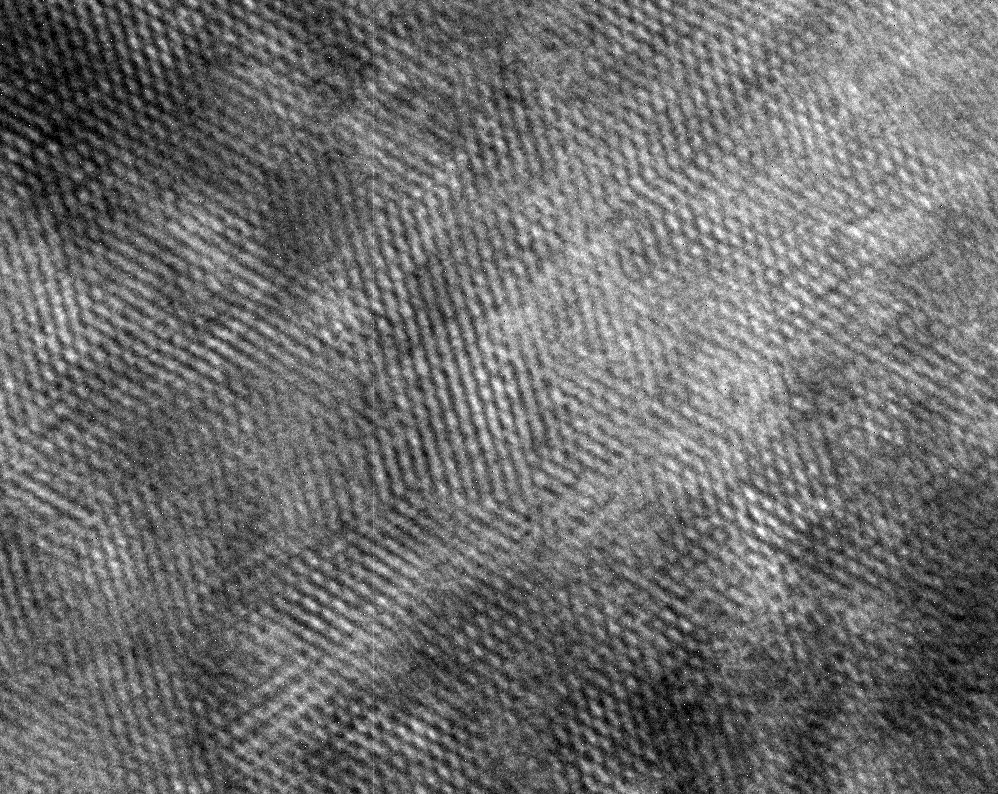


5 nm

3.52 Å Anatase (101)

3.25 Å

Rutile (110)

3.52 Å

Anatase (101)

3.25 Å

Rutile (110)

(d)

**Amorphous layer**

**introduced by hydrogenation**

1 nm


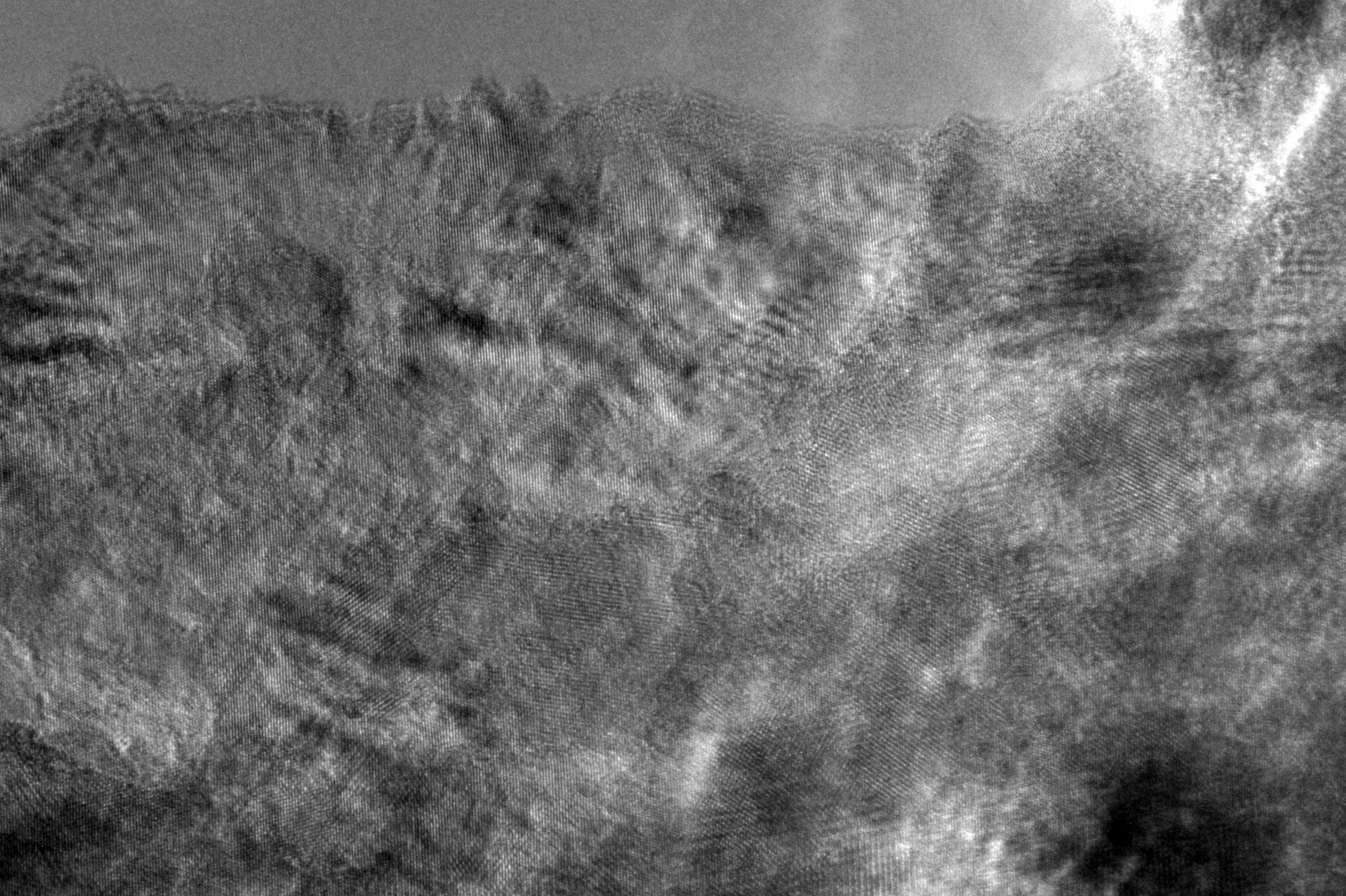

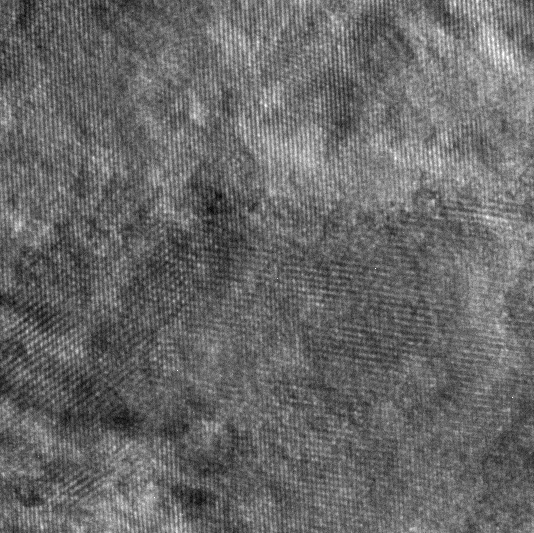


3.25 Å

Rutile (110)

**Lattice**

**disorder**

2.38 Å

Anatase(004)

3.25 Å

Rutile (110)

10 nm

5 nm

(c)

**Figure. S3** Typical TEM image of (a) H@TNAs-3 and (b) H@TNAs-4, HR-TEM image of (c) H@TNAs-2 and (d) H@TNAs-4

*Supercapacitive performances of as-prepared H@TNAs*


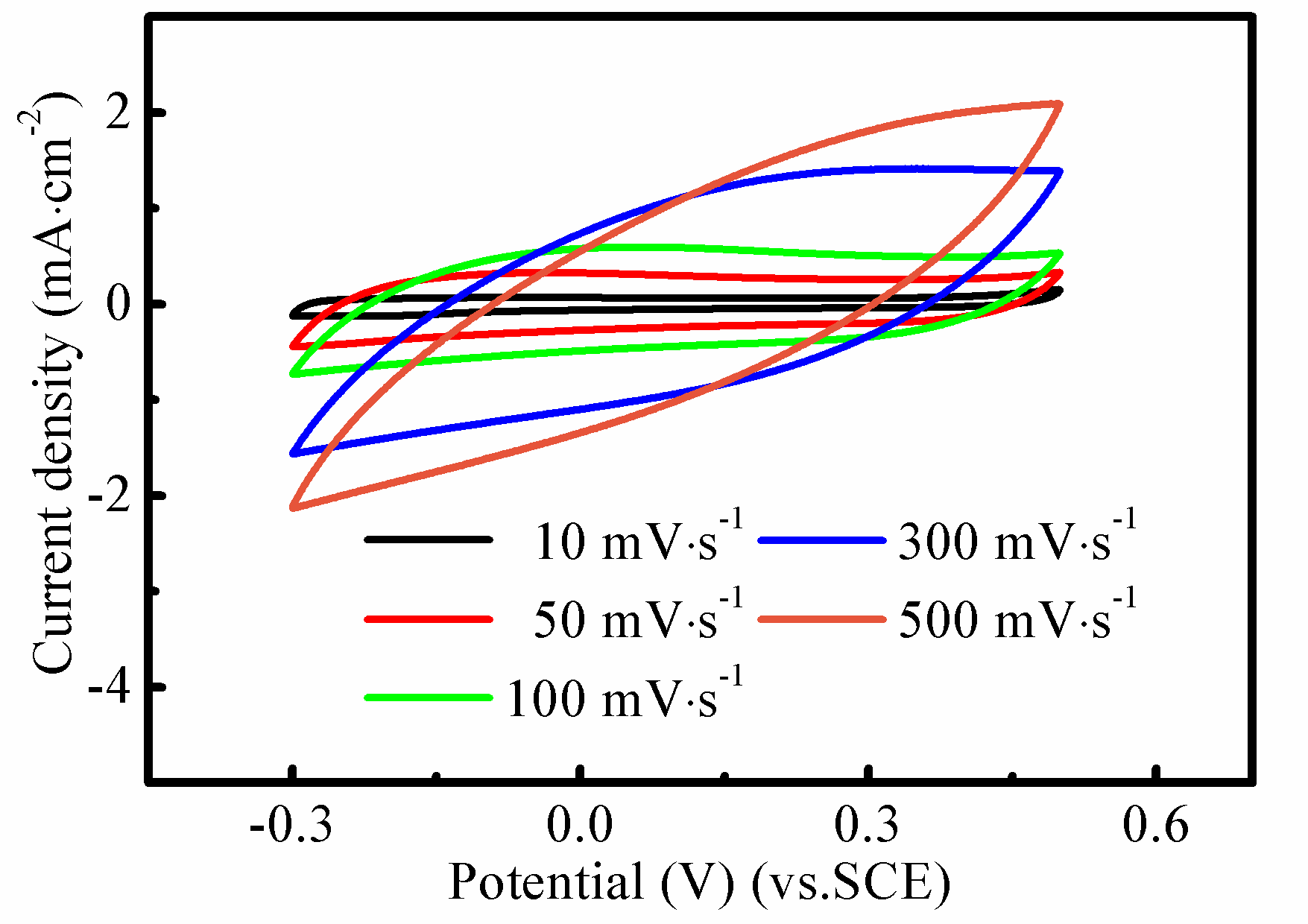

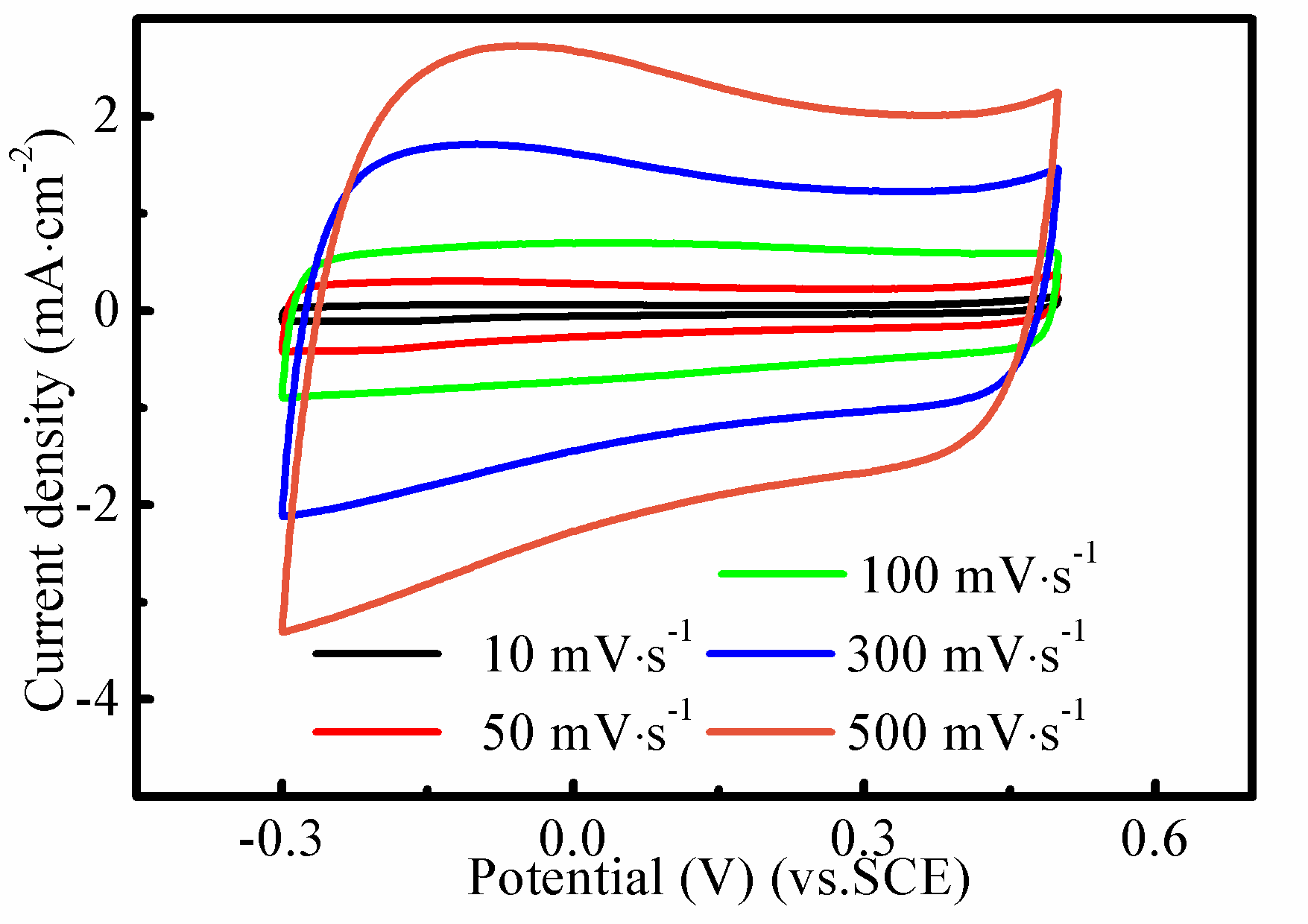

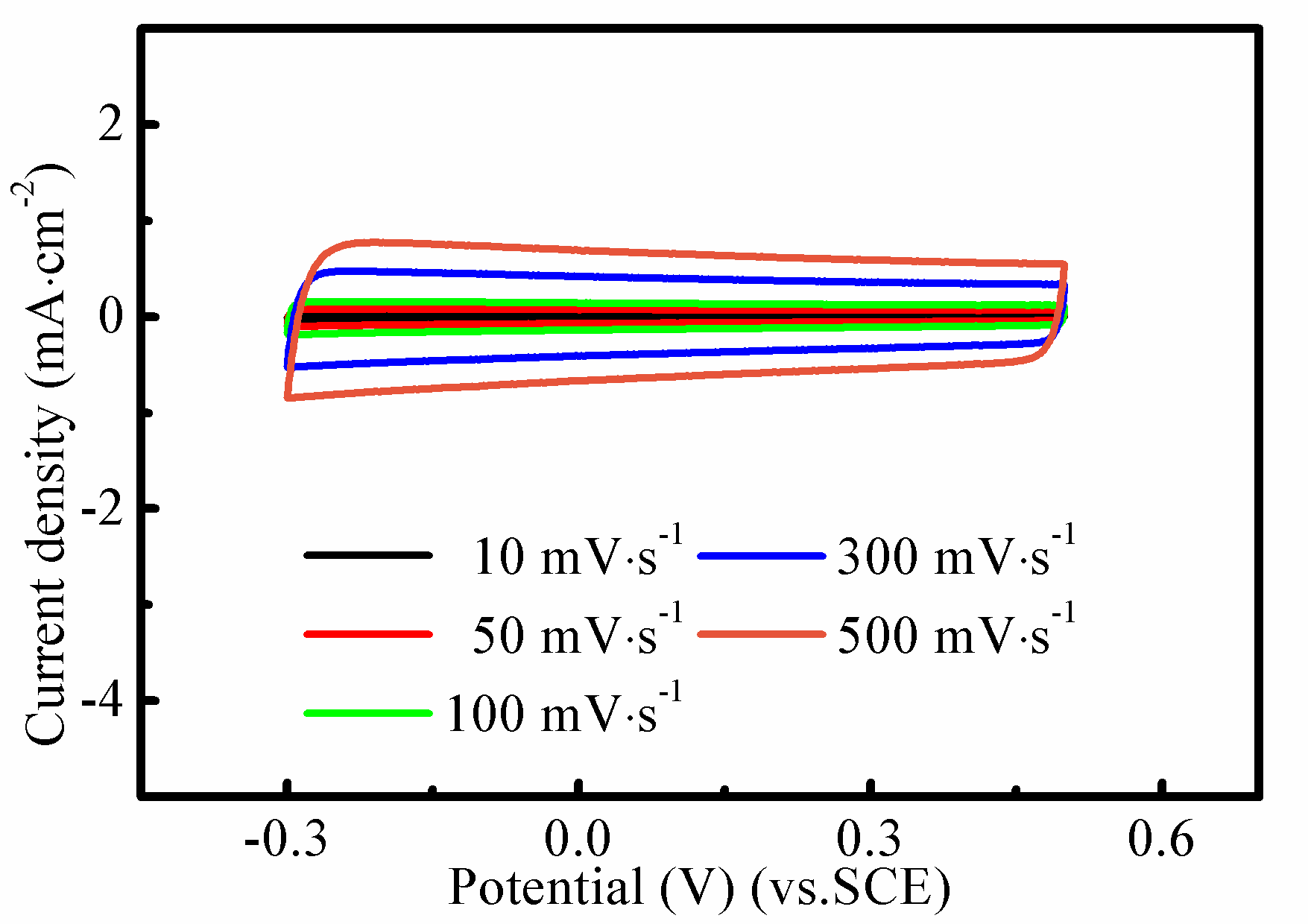

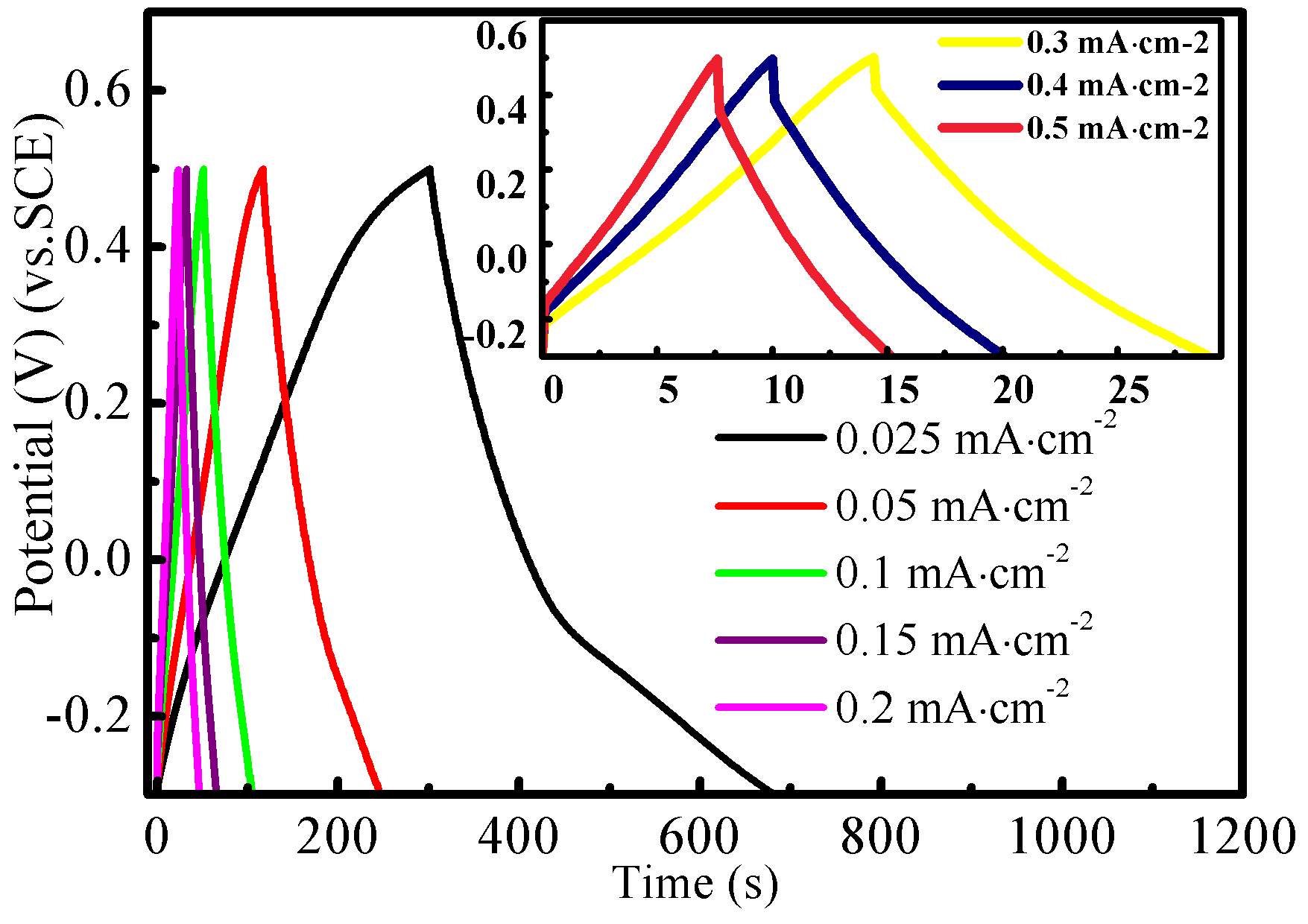

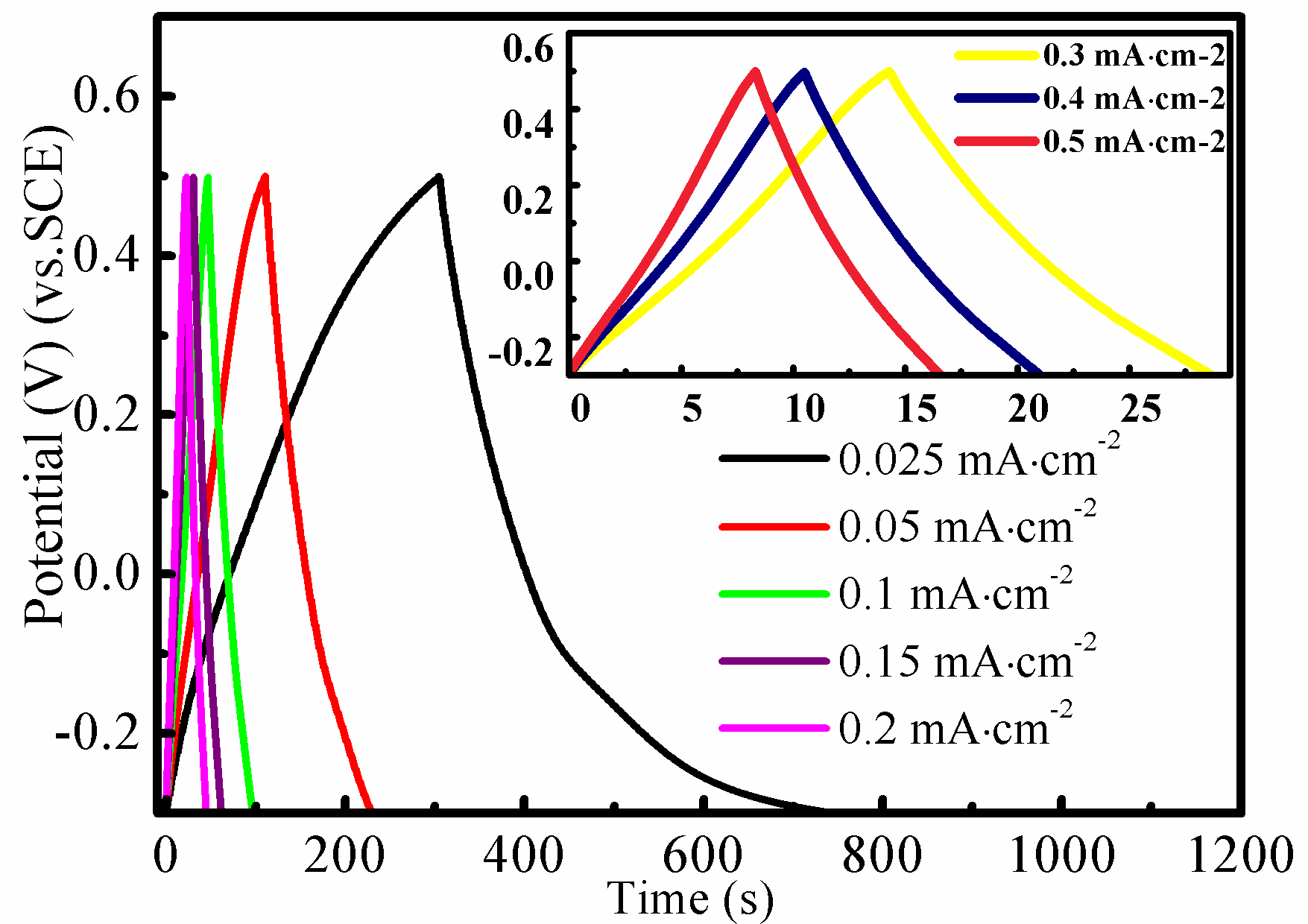

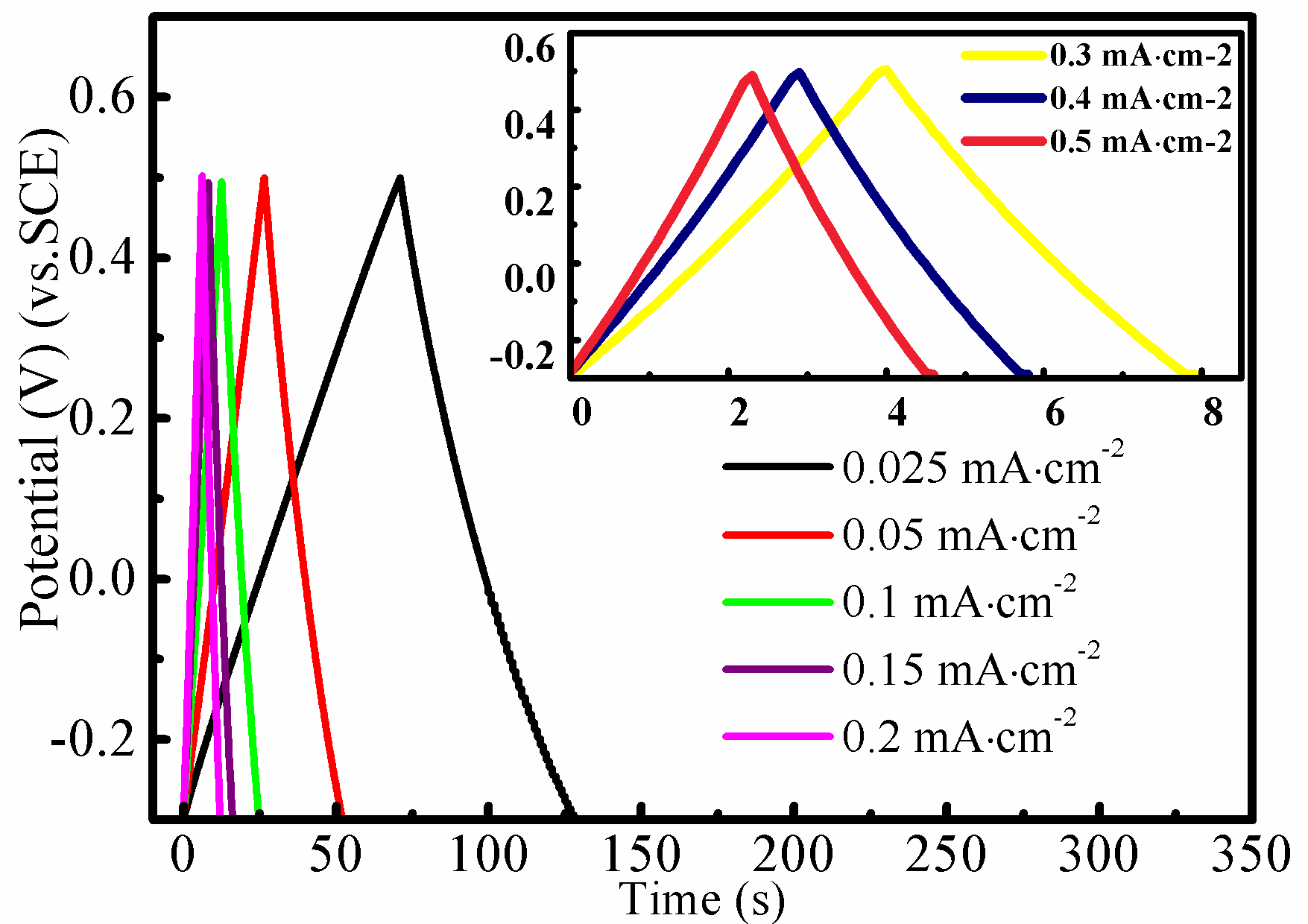


(a)

(b)

(c)

(d)

(e)

(f)

**Figure. S4** CV curves collected at different scan rates ranging from 10 to 500 mV·s^-1^: (a) H@TNAs-2, (b) H@TNAs-3 and (c) H@TNAs-4. Galvanostatic charge/discharge curves at various current densities ranging from 0.025 to 0.5 mA·cm^-2^, inset is the enlargement of the galvanostatic charge/discharge curves at higher current densities: (d) H@TNAs-2, (e) H@TNAs-3 and (f) H@TNAs-4.

**Table. S2** Comparison of the results of some oxygen-deficient TNAs with random orientation in the previous literature.

| Samples | Synthesis | Electrolyte | Specific capacitance (mF·cm^-2^) | Tube length (μm) | (mF·cm^-2^·μm^-1^) | Ref |
| --- | --- | --- | --- | --- | --- | --- |
| H-TiO_2_ NTAs | Heat-treated in hydrogen atmosphere | 0.5 M Na_2_SO_4_ | 3.24 | - |  | [1] |
| rTNAs | NaBH_4_ reduce | 0.5 M Na_2_SO_4_ | 23.24 | 17.5 | 1.328 | [2] |
| TiO_2_-H | Electrochemical doping | 2 M Li_2_SO_4_ | 20.08 | 15 | 1.339 | [3] |
| Electrochemically Self-Doped TiO_2_ | Electrochemical doping | 0.5 M Na_2_SO_4_ | 1.84 | 15 | 0.122 | [4] |
| H@TNAs-1 | Electrochemical doping | 0.5 M Na_2_SO_4_ | 20.86 | 8.38 | 2.49 | This work |
| H@TNAs-2 | Electrochemical doping | 0.5 M Na_2_SO_4_ | 28.23 | 8.52 | 3.31 | This work |
| H@TNAs-3 | Electrochemical doping | 0.5 M Na_2_SO_4_ | 24.99 | 8.68 | 2.88 | This work |

**Table. S3** Equivalent series resistance of as-prepared H@TNAs.

| Samples | H@TNAs-1 | H@TNAs-2 | H@TNAs-3 | H@TNAs-4 |
| --- | --- | --- | --- | --- |
| ESR | 1.5 Ω | 36 Ω | 6.5 Ω | 5.5 Ω |

The equivalent series resistances (ESR) of as-prepared H@TNAs were also calculated by the followed equation[5]:

$R=\frac{V_{\text{c}h\arg e}-V_{disch\arg e}}{2I}$ (1)

Where *V_charge_* is the voltage of the cell at the end charge, *V_discharge_* is the voltage of the cell at the starting discharge and I is the absolute value of the charge/discharge current.

*Results of EIS measurement*

**Table. S4** Fitting parameters of the equivalent circuit for the Nyquist plots

| Samples | R_s_(Ω) | CPE_1_,Yc  (S·s^n^ ) | n_1_ | R_1_(Ω) | CPE_2_,Yc  (S·s^n^ ) | | n_2_ | R_2_(Ω) | χ^2^ |
| --- | --- | --- | --- | --- | --- | --- | --- | --- | --- |
| H@TNAs-1 | 3.101 | 0.01875 | 1 | 2.895×10^4^ | 0.122 | 0.8009 | | 0.3039 | 6.93×10^-4^ |
| H@TNAs-2 | 3.126 | 0.02582 | 0.9631 | 3.16×10^4^ | 7.95×10^-6^ | 0.7993 | | 29.28 | 2.02×10^-3^ |
| H@TNAs-3 | 3.255 | 0.01830 | 0.9799 | 9050 | 1.312 | 0.6188 | | 2.82 | 6.45×10^-4^ |
| H@TNAs-4 | 3.467 | 0.00488 | 1 | 1.441×10^4^ | 0.1651 | 0.5322 | | 1.156 | 8.73×10^-4^ |

**Table. S5** The calculations of C

| Samples | H@TNAs-1 | H@TNAs-2 | H@TNAs-3 | H@TNAs-4 |
| --- | --- | --- | --- | --- |
| C (mF·cm^-2^) | 18.75 | 25.82 | 18.30 | 4.88 |

The double layer capacitor (*C*) formed on the interface between electrolyte and electrode also can be calculated based on the fitting parameters of EIS. *C* is calculated by using the following equation[6]:

$C={Y_{c}}^{\frac{1}{n}}\times{R_{1}}^{(\frac{1-n}{n})}$ (2)

Where, *C* is the fitting value of the capacitance of the double layer capacitor formed on the interface between electrolyte and electrode, *Y_c_* is the time coefficient, n is served as an index to evaluate the deviation from the ideal electric double layer capacitor, who is in a range of 0 and 1 (when n=1, a CPE simplifies a capacitor) depending on the surface structure of electrodes, R_1_ is the polarization resistance. The results are close to the values obtained in the above galvanostatic charge-discharge tests.

*Cyclic performance*


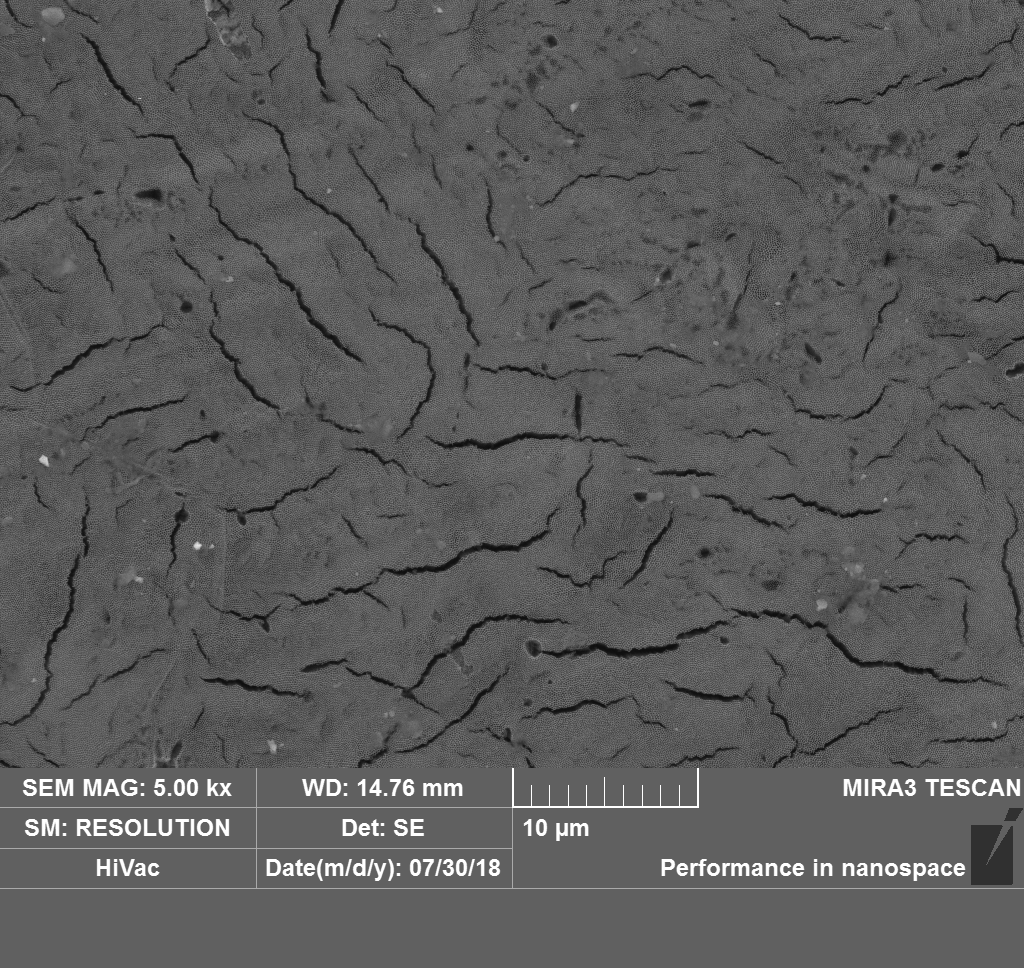

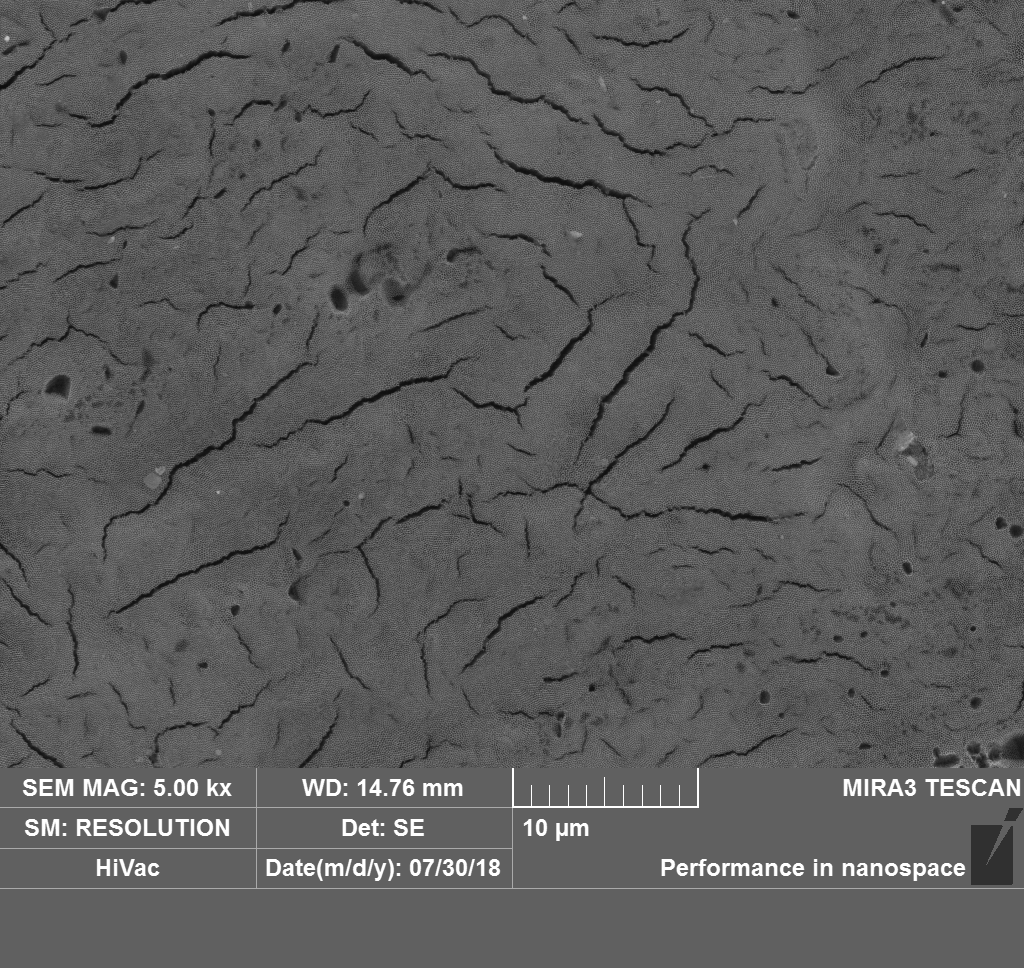

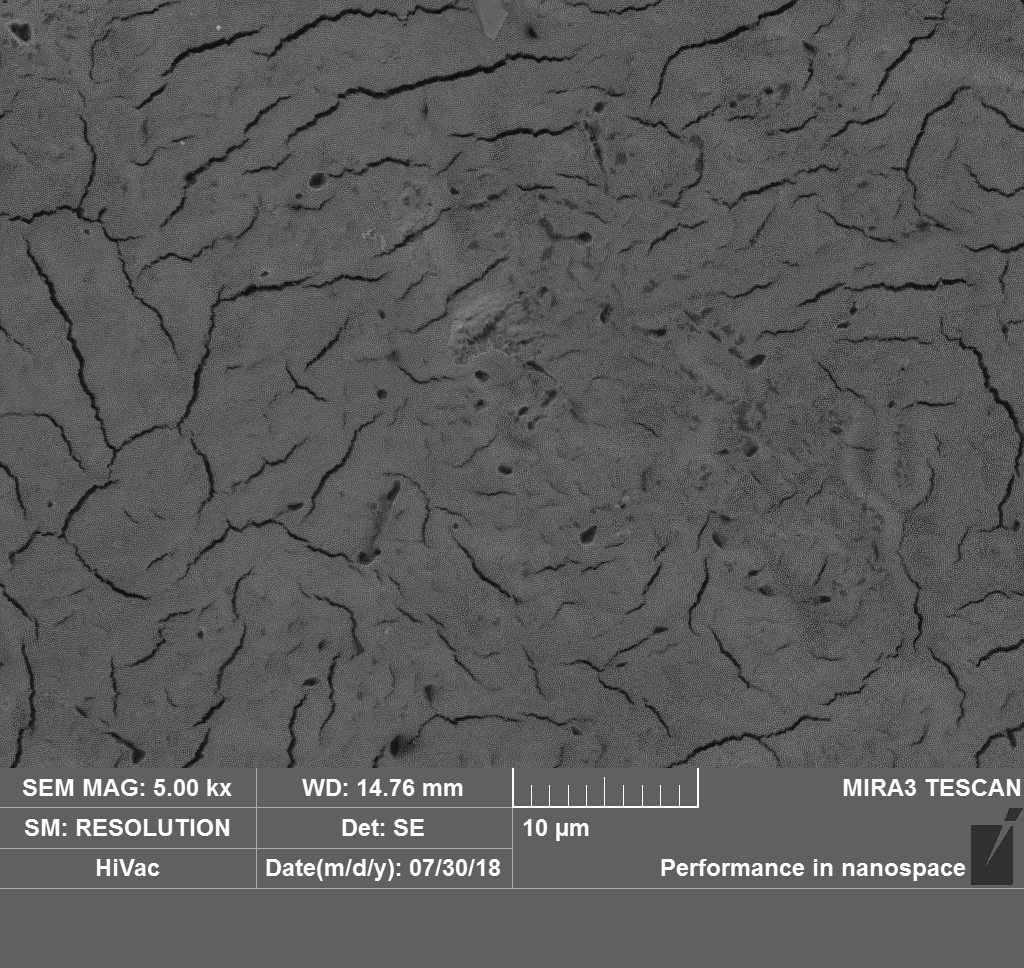

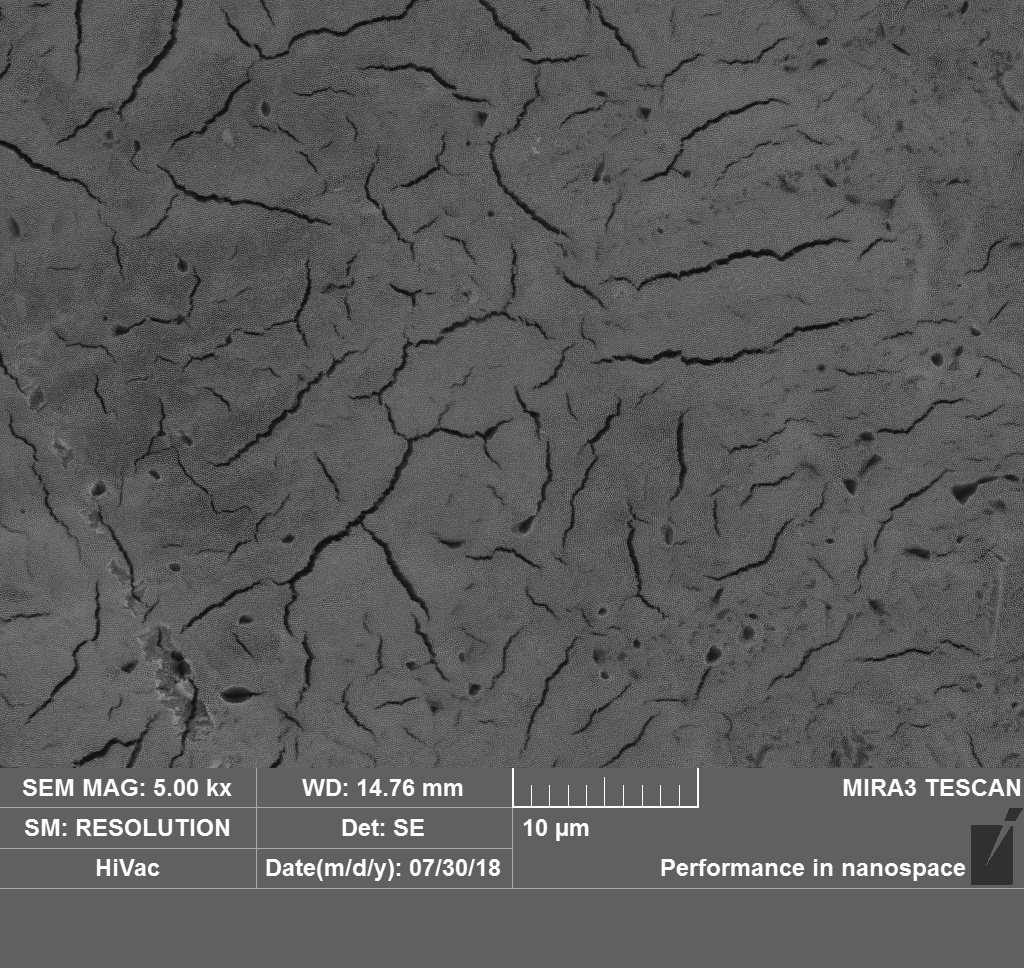

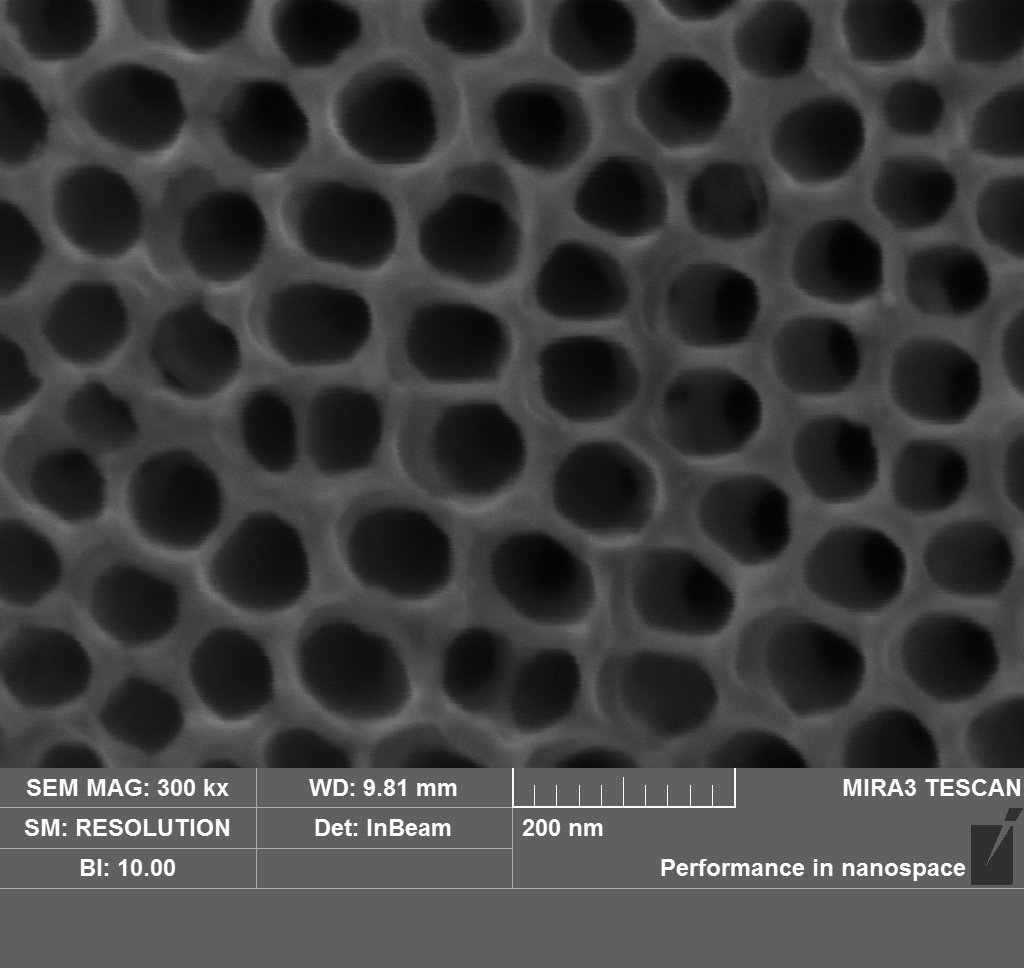

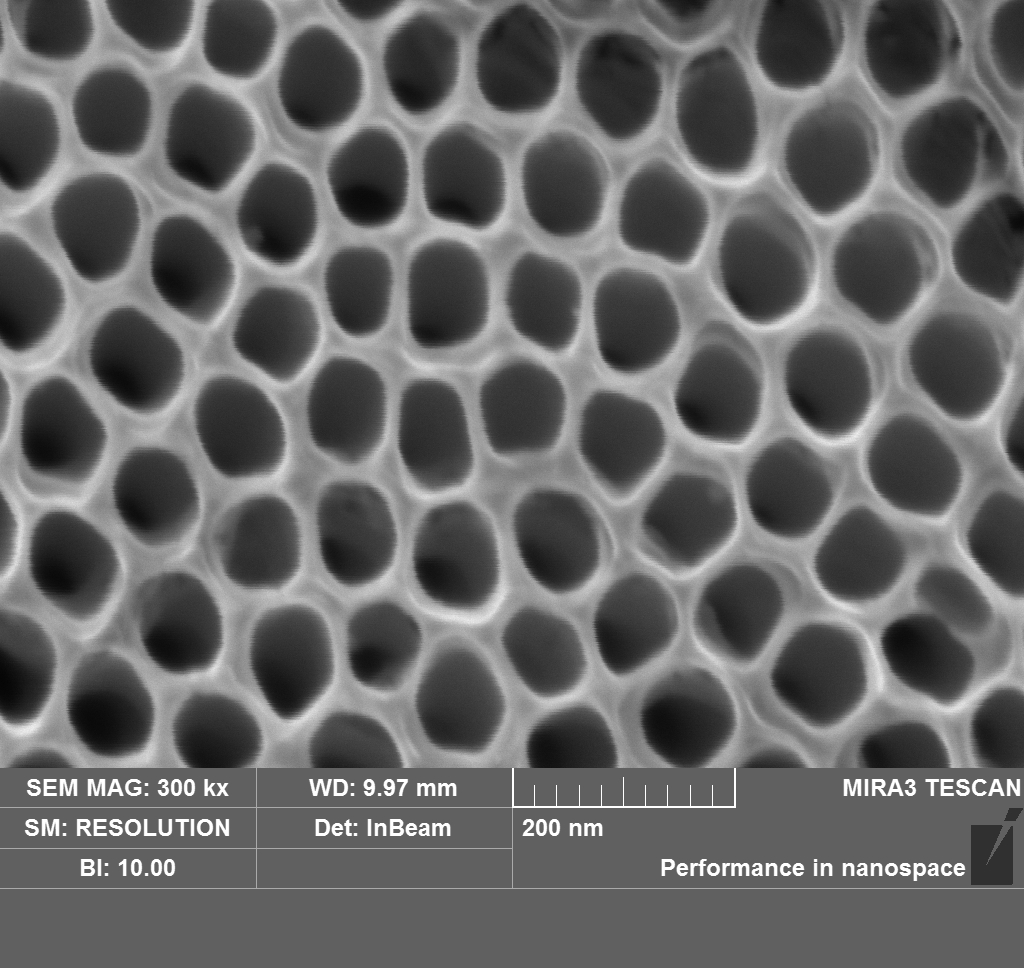

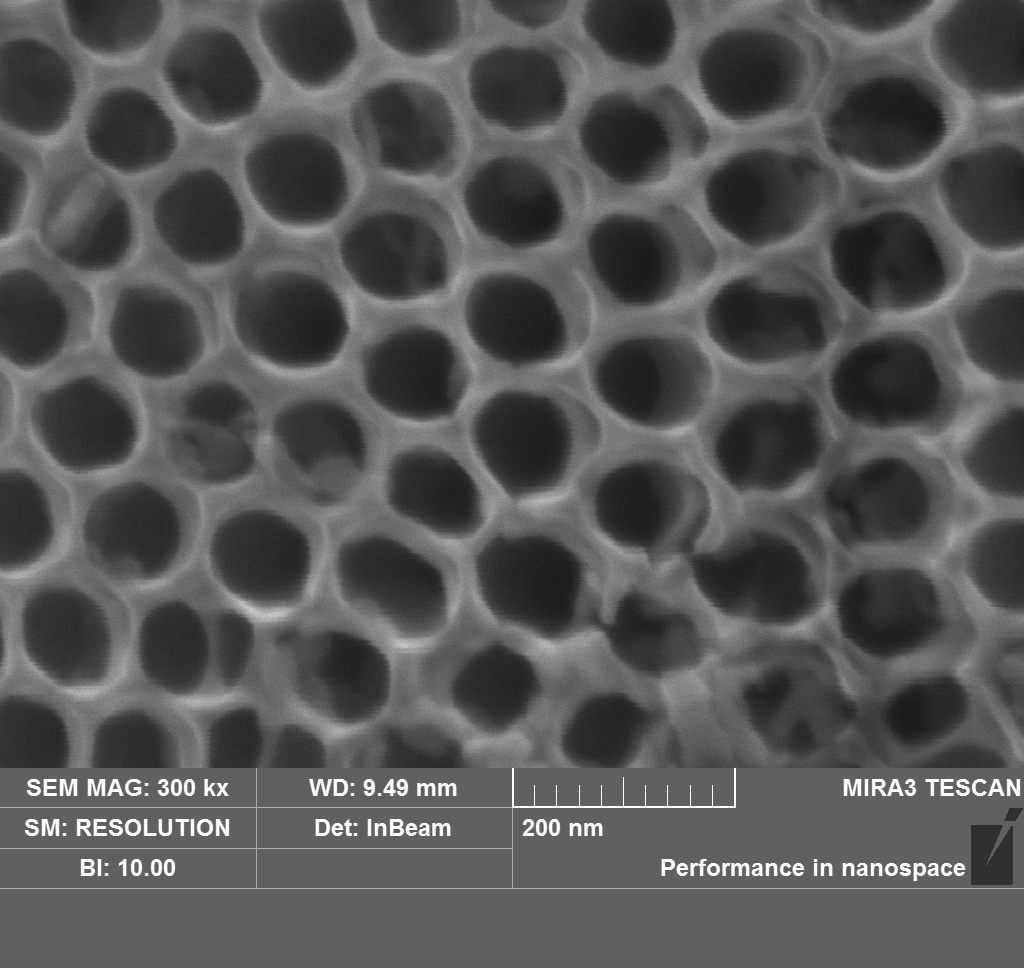

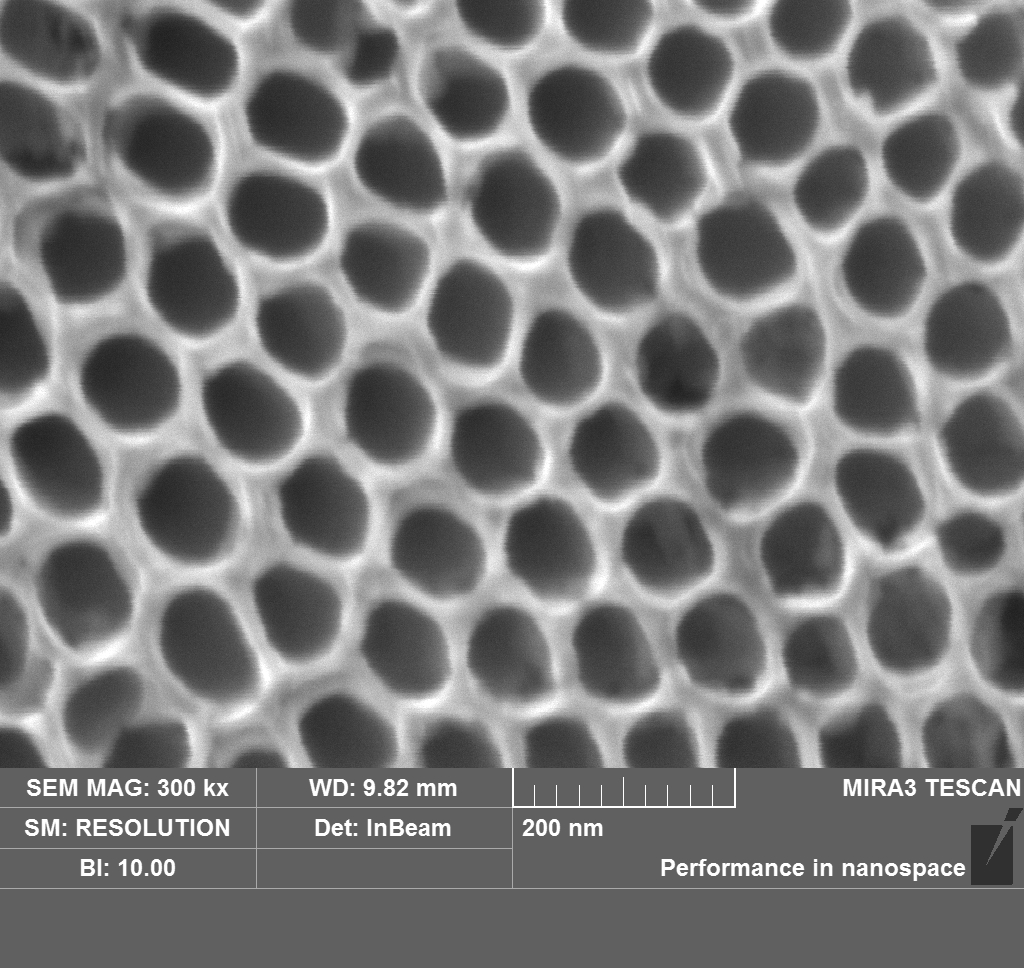


10 μm

10 μm

10 μm

10 μm

200 nm

200 nm

200 nm

200 nm

a

b

c

d

**Figure. S5** Surface morphology of each sample after 5000 cycles: (a) H@TNAs-1, (b) H@TNAs-2 and (c) H@TNAs-3 and (d) H@TNAs-4.

Such a superior cyclic performance mainly benefited from the outstanding structural stability of TiO_2_ and the energy storage mechanism which is just a physical electrostatic adsorption process during fast charge/discharge. There were no redox reactions in charge/discharge process, thus there were less irreversible electrochemical reactions, coupled with the stable structure which ensured the electrode was not damaged during cycling test (seen in Figure. S5(a) to Figure. S5(d)). Hence, each sample possessed a relatively small irreversible capacitance.

**Table. S6** Comparison of the discharge specific areal capacitances before and after 5000 cycles.

| Samples | H@TNAs-1 | H@TNAs-2 | H@TNAs-3 | H@TNAs-4 |
| --- | --- | --- | --- | --- |
| Cbefore cycling (mF·cm^-2^) | 18.12 | 16.21 | 18.01 | 5.30 |
| Cafter cycling (mF·cm^-2^) | 17.03 | 15.08 | 17.10 | 5.04 |
| retention rates | 94% | 93% | 95% | 95% |

*Energy densities and power densities*

**Table. S7** Energy densities and power densities of as-prepared H@TNAs.

| Samples | H@TNAs-1 | H@TNAs-2 | H@TNAs-3 | H@TNAs-4 |
| --- | --- | --- | --- | --- |
| Energy density (mWh·cm^-2^) | 5.81 | 5.28 | 5.74 | 1.68 |
| Power density (W·cm-2) | 1.55 | 1.31 | 1.45 | 1.54 |

The energy density and the power density of each sample were calculated at 0.3 mA·cm^-2^ using the equation (3) and (4)[7], where C was the specific capacitance of H@TNAs, △V was the potential window and t was the discharge time.

$E=\frac{1}{2}C(\Delta V)^{2}$ (3)

$P=\frac{E}{t}$ (4)

## Reference

1. Lu X, Wang G, Zhai T, Yu M, Gan J, Tong Y, et al. Hydrogenated TiO_2_ nanotube arrays for supercapacitors. Nano Letters. 2012;12(3):1690-1696.

2. Zhang J, Wang Y, Wu J, Shu X, Yu C, Cui J. Remarkable supercapacitive performance of TiO_2_ nanotube arrays by introduction of oxygen vacancies. Chemical Engineering Journal. 2017;313:1071-1081.

3. Wu H, Li D, Zhu X, Yang C, Liu D, Chen X, et al. High-performance and renewable supercapacitors based on TiO_2_ nanotube array electrodes treated by an electrochemical doping approach. Electrochimica Acta. 2014;116:129-136.

4. Minguzzi A, Sánchez‐Sánchez CM, Gallo A, Montiel V, Rondinini S. Evidence of facilitated electron transfer on hydrogenated self-doped TiO_2_ nanocrystals. Chemelectrochem. 2015;1(8):1415-1421.

5. Laheäär A, Przygocki P, Abbas Q, Béguin F. Appropriate methods for evaluating the efficiency and capacitive behavior of different types of supercapacitors. Electrochemistry Communications. 2015;60:21-25.

6. Harrington SP, Devine TM. Relation between the semiconducting properties of a passive film and reduction reaction rates. Journal of the Electrochemical Society. 2009;156(4):C154-C9.

7. Mai LQ, Minhas-Khan A, Tian X, Hercule KM, Zhao YL, Lin X, et al. Synergistic interaction between redox-active electrolyte and binder-free functionalized carbon for ultrahigh supercapacitor performance. Nature Communications. 2013;4:2923.
